# Carbapenem-resistant *Escherichia coli* from shrimp and salmon available for purchase by consumers in Canada: a risk profile using the Codex framework

**DOI:** 10.1017/S0950268822001030

**Published:** 2022-06-29

**Authors:** Daleen Loest, F. Carl Uhland, Kaitlin M. Young, Xian-Zhi Li, Michael R. Mulvey, Richard Reid-Smith, Lauren M. Sherk, Carolee A. Carson

**Affiliations:** 1 Centre for Food-borne, Environmental and Zoonotic Infectious Diseases, Public Health Agency of Canada, Guelph, Ontario, Canada; 2 Veterinary Drugs Directorate, Health Products and Food Branch, Health Canada, Ottawa, Ontario, Canada; 3 National Microbiology Laboratory, Public Health Agency of Canada, Winnipeg, Manitoba, Canada

**Keywords:** Canada, carbapenem resistance, carbapenems, Codex, *Escherichia coli*, risk profile, salmon, seafood, shrimp

## Abstract

Resistance to carbapenems in human pathogens is a growing clinical and public health concern. The carbapenems are in an antimicrobial class considered last-resort, they are used to treat human infections caused by multidrug-resistant Enterobacterales, and they are classified by the World Health Organization as ‘High Priority Critically Important Antimicrobials’. The presence of carbapenem-resistant Enterobacterales (CREs) of animal-origin is of concern because targeted studies of Canadian retail seafood revealed the presence of carbapenem resistance in a small number of Enterobacterales isolates. To further investigate this issue, a risk profile was developed examining shrimp and salmon, the two most important seafood commodities consumed by Canadians and *Escherichia coli*, a member of the Enterobacterales order. Carbapenem-resistant *E. coli* (CREc) isolates have been identified in shrimp and other seafood products. Although carbapenem use in aquaculture has not been reported, several classes of antimicrobials are utilised globally and co-selection of antimicrobial-resistant microorganisms in an aquaculture setting is also of concern. CREs have been identified in retail seafood purchased in Canada and are currently thought to be uncommon. However, data concerning CRE or CREc occurrence and distribution in seafood are limited, and argue for implementation of ongoing or periodic surveillance.

## Introduction

Antimicrobial-resistant bacteria are a global public health concern. To assess the risk to human health from foodborne AMR hazards, a risk analysis is a valuable tool in the risk manager's armamentarium [1]. One of the initial steps in a risk analysis is the compilation of information in a risk profile, as described in the ‘Guidelines for Risk Analysis of Foodborne Antimicrobial Resistance’, adopted by the Codex Alimentarius Commission (herein denoted the ‘Codex Guidelines’) [1]. A risk profile can help identify subsequent risk analysis steps, ranging from immediate (and/or provisional) risk management decisions, launching of a full qualitative or quantitative risk assessment, identifying the need for additional data gathering before making a preliminary decision or maintaining the *status quo.*

Using the Codex language, an AMR food safety issue is a combination of an AMR hazard of concern (bacteria or gene), an antimicrobial agent and a food commodity where the hazard is found [1]. The AMR food safety issue described in this risk profile is carbapenem-resistant *Escherichia coli* (CREc) originating from salmon and shrimp available for purchase by consumers in Canada.

Carbapenems are of interest because of their importance in the treatment of severe human disease caused by multidrug-resistant (MDR) pathogens and the presence of CREc in the aquatic/aquaculture environment in recent literature[2–4]. Although carbapenem use is not currently reported in aquaculture, co-selection and antimicrobial resistance gene (ARG) acquisition coding for carbapenem resistance are of utmost concern. *E. coli* is a commensal of humans and other mammals, can be a serious pathogen and has been found as a contaminant in animals and food. Its usefulness in detecting ARG flux in the agrifood industry has been explored in several national AMR surveillance programmes [5,6].

Finally, the food commodities under consideration in this risk profile were salmon and shrimp as they are the most commonly consumed seafood products in Canada [7]. The majority of shrimp consumed in Canada are imported products, whereas salmon are principally domestically produced. This is of importance when considering AMR sources and control options within Canada. Carbapenem resistance genes have already been identified in retail seafood in Canada, but their occurrence and distribution remain unknown.

The objectives of this paper were to evaluate this specific AMR food safety issue to provide information to risk managers concerning the current state of knowledge as to the AMR hazard/risk potential and recommend further action. To our knowledge, this is the first time that this specific AMR food safety issue for seafood has been described and evaluated in a formal risk context utilising the Codex Guidelines.

## Materials and methods

The risk profile described in the results of this paper is organised following the Codex guidelines and recommendations for foodborne AMR risk analysis. The data were collated and reported utilising the suggested elements for inclusion in the Codex Guidelines' Appendix 1: Elements for Consideration in a Foodborne Antimicrobial Resistance Risk Profile [1]. Data sources included relevant seafood studies from the Canadian Integrated Program for Antimicrobial Resistance Surveillance (CIPARS), Fisheries and Oceans Canada, peer-reviewed literature, grey literature, expert opinion and demographic information from Statistics Canada and Agriculture and Agri-Food Canada. The collated information pertains to Canadian data and the Canadian context, unless indicated otherwise. If non-Canadian data were used (i.e. when there were identified gaps in Canadian data), this was acknowledged in the data quality evaluation.

Where applicable, the data sources for each section were assessed for data quality using the following criteria: applicability of the data within a Canadian context based on the location of information collected, type of study (e.g. surveillance *vs.* empirical information) and year of data collection. Scores across the subsections of information were averaged to provide an overall measure of data quality ranging from 0 to 10. Higher scores indicate better data for the evaluation of the current risk.

To help advise policy makers, each major section of the risk profile was also subjectively categorised into ‘levels of concern’ (1 = lowest concern; 3 = highest concern), considering the significance of the antimicrobial in question, the existence and quality of available data and the necessity of action to solidify existing data or fill data gaps to ensure informed decisions [8]. Further details are provided in Supplementary Material, Excel file S1 and Table S1.

Where appropriate and informative, or when *E. coli* specific data were lacking, data for other Enterobacterales (including carbapenem-resistant Enterobacterales (CRE) and carbapenemase-producing Enterobacterales (CPE)) and aquatic bacterial species (*Aeromonas* spp. and *Vibrio* spp.) were included, as these Gram-negative organisms can share similar mechanisms of resistance.

## Results (headings as per the Codex Guidelines)

### Description of the AMR food safety issue

Enterobacterales demonstrating resistance to carbapenems have been isolated from seafood products and the aquaculture environment in Canada and other countries [4,9,10]. *E. coli* was chosen as it is a commensal of humans and other mammals, and can be a serious human pathogen. It has been identified in all sectors of the aquaculture farm to fork environment (culture, harvesting, processing and retail) and isolates resistant to carbapenems have been identified in shrimp and other seafood products [3, 4,11,12]. *E. coli* is also an important model organism for AMR surveillance/detection due to its capacity for genetic promiscuity, facilitating ARG exchanges.

The antimicrobials under scrutiny are the carbapenems, considered last-resort antimicrobials and classified by the World Health Organization (WHO) as ‘High Priority Critically Important Antimicrobials’ [13] and by Health Canada as ‘Category I – Antimicrobials of Very High Importance’ [14]. Carbapenems can be used to treat human infections caused by MDR Enterobacterales, for which few treatment alternatives exist, and such infections may result from transmission of Enterobacterales, including *E. coli*, from non-human sources [13].

The Canadian seafood marketplace is diverse in terms of product, country-of-origin and production method. Finfish and shellfish available at the retail-level can be of imported or domestic origin, wild harvested or grown in aquaculture facilities. Salmon and shrimp were the products examined here. The vast majority of shrimp consumed by Canadians are imported from Asian countries, whereas the majority of Canadian retail salmon sold are domestically grown. Canada has strict regulations concerning antimicrobial use (AMU) in aquaculture, which ensure safe and healthy products. As the regulatory environment and AMU in aquaculture can vary between countries, this takes on additional importance in the context of increasing worldwide exchange of agriculture products. Unlike risk profiles regarding food from terrestrial animals, the aquatic environment can act as a reservoir and probable source, as well as a receiver of ARGs from terrestrial effluent [15–17]. Therefore aquaculture risk profiles need to consider ARG acquisition by bacteria as a consequence of AMU in aquatic species, exposure to terrestrial contamination and the environmental resistome. Aquaculture production is found at the confluence of these three elements, which may shape and define the development, propagation and transmission of ARGs to the human population.

### Information on the AMR microorganism(s) and/or determinant(s)

#### Characteristics of the identified foodborne microorganism(s)

*Sources and transmission routes*. The principle route of transmission of CREc to humans considered in this risk profile is via consumption of contaminated salmon and shrimp. Although *E. coli* is not considered a commensal or pathogen of aquatic hosts, they are frequently encountered in studies examining bacterial flora and AMR in seafood products [18–20]. However, foodborne disease outbreaks attributed to *E. coli* in seafood are considered infrequent. An analysis of publicly available reports indicated that *E. coli* was associated with 0.8% of the total foodborne outbreaks due to seafood (*N* = 277) reported internationally between 1988 and 2007 [21]. An outbreak of enterotoxigenic *E. coli* associated with consumption of shrimp and attributed to poor food-handling practices and infected food-handlers in a Nevada sushi restaurant and another concerning *E. coli* O157 in salted salmon roe in Japan were identified in the literature [22,23].

*Pathogenicity, virulence and linkage to resistance of particular strains*. *E. coli* strains can possess pathogenicity and virulence elements and cause intestinal and extra-intestinal diseases, including life-threatening complications in people [24,25]. Enteropathogenic *E. coli* have been found to contain a diversity of mobile plasmids encoding virulence factors such as secretion systems mediating bacterial adherence to the host epithelial cells and heat-labile/heat-stable toxin production in addition to ARGs (e.g. for spectinomycin-streptomycin, sulphonamide and tetracycline resistance) [24,26–28]. Pathogenic, and particularly Shiga-toxigenic *E. coli* strains have been identified in seafood products and their production environment including shellfish, raw and ready-to-eat fish and retail shrimp [29–37]. The coexistence of virulence/pathogenicity genes and ARGs has been demonstrated in several studies of *E. coli* isolated from the aquatic environment and seafood [38–45].

Carbapenem resistance in *E. coli* is typically mediated by plasmids or other mobile elements encoding carbapenemase genes such as *bla*_KPC_, *bla*_NDM_ and *bla*_OXA−48−like_ [46–48]. CREc phenotypes isolated from the aquatic environment or seafood have been reported [49]. In a study of Brazilian shrimp farms, almost 86% of *E. coli* isolates from pond sediment, water and shrimp demonstrated resistance or intermediate resistance to imipenem [11]. Specific CREc carbapenemase ARGs have also been identified in both the aquatic environment (KPC-2, VIM-1, VIM-34 and IMP-8) and seafood (VIM-1, NDM-1, NDM-5) [3,4,47,50,51]. In a retail seafood study by Roschanski *et al*. [3], the VIM-1 carbapenemase gene and 12 other resistance genes (associated with resistance to *β*-lactams, aminoglycosides, chloramphenicol, macrolides, fluoroquinolones and sulphonamides/trimethoprim) were shown to be harboured by a class I integron-containing plasmid from an *E. coli* (sequence type ST10) isolated from a Venus clam [3]. The plasmid also contained the *gad* and *iss* virulence genes with *gad* being among the core group of virulence genes known to be present in this common sequence type of human and food animal sources [52].

*Growth, survivability and inactivation in foods (e.g. D-value, minimum pH for growth, etc.) of foodborne AMR microorganisms in the food commodity production to consumption continuum*. Contamination of seafood by *E. coli* can occur at multiple points along the production-to-consumption continuum, from the aquatic and culture environment, to processing, retail and food preparation [53,54]. A comparison of enterohaemorrhagic *E. coli* (EHEC) from human and animal sources demonstrated the ability of this organism to survive in the aquatic environment for variable periods depending upon water temperatures and physicochemical variables [55]. This capacity to survive in the aquatic environment may facilitate ARG exchange and contamination of the seafood production chain.

The US Food and Drug Administration (FDA) has published limits of different physicochemical properties of seafood at which bacterial growth can be sustained including temperature, pH and salinity [56]. Though none of these variables are known to preferentially affect the frequency of carbapenem resistance in *E. coli*, they impact directly the prevalence of *E. coli* in seafood. Additionally, the values published by the FDA concern pathogenic *E. coli*, which may differ from commensal or non-pathogenic strains.

Lower and upper temperature limits described for pathogenic *E. coli* growth in seafood are 6.5 and 49.4 °C, respectively [56]. At higher temperatures, survival of *E. coli* following thermal stress (cooking) is similar in seafood to other animal products including beef, chicken and turkey [57,58]. However, at the lower temperature ranges cited, bacterial growth may occur. Cwiková noted that *E. coli* concentrations in salmon samples increased similarly following 2 days of storage at 4 or 8 °C [59].

The upper and lower limits of pH for pathogenic *E. coli* growth, according to the FDA, are 4 and 10, respectively. The pH values for salmon and shrimp flesh occupy a narrow range from 6.42 to 7.18, and 6.42 to 6.8 respectively, well within the FDA's range and therefore conducive to *E. coli* growth [60–62].

Modulation of water content (water activity – A_W_) and salinity (water phase salt – WPS) is important for prolonging shelf-life and ensuring seafood quality, especially for dried, smoked and salted fish products. An A_W_ level below 0.85 and a WPS value of 6.5% are considered limiting for bacterial growth [56,63].

*Distribution, frequency and concentrations of the AMR hazard(s) in the food chain*. Several researchers have investigated the presence of *E. coli* at various points along the seafood production-to-consumption continuum. Although *E. coli* is not considered normal bacterial flora in the aquaculture farming/aquatic environment, exposure may result from the water source or culture unit contamination (e.g. manuring, integrated farming or terrestrial anthropogenic/agricultural effluents). Studies examining *E. coli* and shrimp farms in Southeast Asia found the prevalence of *E. coli* varied widely, ranging from 3% to 21% and 5% to 89% in water and sediment samples respectively, depending on the culture and sampling scheme [64,65]. Dewanti-Hariyadi also identified high concentrations in farmed shrimp at four sites sampled in Western Indonesia ranging from 4.4% to 5.7% log_10_CFU/g [66].

*E. coli* has been reported among samples of shrimp or prawn at the farm-level, and its prevalence has been found to vary widely [64,65,67,68]. In a study of six countries (three in Asia and one each in Central America, North America and the Pacific Islands) that best represent the shrimp aquaculture industry, 6–88% of sampled shrimp demonstrated the presence of *E. coli* at concentrations of >10 CFU/g [64]. Other studies examining contamination of shrimp found that farmed and wild caught shrimp did not differ appreciably with *E. coli* concentration values between <1–10 000 and <1–2239 CFU/g, respectively [65,66,69].

Between farm and retail (processing centres, depots, landing centres), *E. coli* prevalence in shrimp sampled in Asian countries varied from just over 1% to as high as 53% [20,68,70–72].

Shrimp sampled at the retail level in the Americas, Asia and Europe yielded an *E. coli* prevalence of 2–40%, with the highest reported in shrimp tail samples purchased at local markets in Brazil [45,66,67,72–77]. In the aforementioned studies, concentrations ranged from 316 CFU/g in German fresh and frozen sushi to as high as 1.2 × 10^5^ CFU/g in Bangladeshian market shrimp. The prevalence of *E. coli* contamination of salmon at the retail level has been reported between 1.5% and 4.8% in the USA, Europe and South America, with concentrations varying from <3–4.6 × 10^2^ to 4.5 × 10^4^ CFU/g in Brazil, Germany and the Czech Republic [59,75–78]. Further details are presented in Supplementary material Table S2.

In Canada, targeted surveillance studies of seafood were undertaken by the Canadian Integrated Program for Antimicrobial Resistance Surveillance (CIPARS) from 2008 to 2016 which examined retail salmon and shrimp. In total, 1061 isolates were identified as *E. coli* out of 2999 samples tested (35%). This included 331 isolates/1361 salmon samples (24%) and 730 isolates/1638 shrimp samples (44.5%). In these studies, *E. coli* found in shrimp was most frequently isolated from imported products, whereas in salmon, those products of domestic or of unknown origin were most often the source of *E. coli* isolates (unpublished data from CIPARS).

Among *E. coli* that have been isolated from the aquatic/aquaculture environment and seafood, phenotypic and genetic resistance to various antimicrobials has been identified, including carbapenems (Supplementary material Table S3). All shrimp sampled in the study by Dos Vieira (2010) yielded *E. coli* isolates resistant to imipenem, and carbapenem ARGs, *bla*_NDM−5_ and *bla*_VIM−1_, have been found in Indian and German retail seafood, respectively [3,4,11,51].

To date, CREc have not yet been identified in *E. coli* in Canadian seafood. However Janecko *et al*. [9] examined 1238 seafood samples imported to Canada from Southeast Asia and found eight isolates of *Enterobacter cloacae* or *Enterobacter aerogenes* harbouring *bla*_IMI−1_, *bla*_IMI−2_ or *bla*_NDM−1_ carbapenemase genes with *bla*_IMI−2_ being plasmid borne, in addition to a novel carbapenemase isolated from a *Vibrio cholerae* isolate named *Vibrio cholerae* Carbapenema-1 (VCC-1) [9,79].

#### Characteristics of the resistance expressed by the AMR microorganism(s) and/or determinant(s)

*Resistance mechanisms and location of AMR determinants*. Among the Enterobacterales (e.g., *E. coli*, *Salmonella* spp.) and aquatic bacteria such as *Aeromonas* spp., *Shewanella* spp. and *Vibrio* spp., resistance to carbapenems is predominantly mediated by the production of carbapenemase *β*-lactamases encoded by chromosomal genes or by plasmids [80,81]. Other mechanisms of resistance, which are typically chromosomal-mediated and include alterations in the target penicillin-binding proteins and reduced drug accessibility (because of porin deficiency and/or elevated drug efflux), are either uncommon or mostly cause low-level reduced carbapenem susceptibility [82,83].

In general, carbapenemases hydrolyse not only carbapenems, but also almost all other *β*-lactams. These enzymes are versatile and consist of Ambler molecular class A serine *β*-lactamases, class B metallo-*β*-lactamases and class D serine OXA *β*-lactamases [80]. The presence of chromosomally-encoded carbapenemases render the microorganisms intrinsically resistant to carbapenems and other *β*-lactams, as observed with IMI enzymes in *E. cloacae* and OXA enzymes in *Acinetobacter bauamannii*. Plasmid-encoded carbapenemases mediate acquired carbapenem resistance in many species of the Enterobacterales order (including *E. coli*, *Salmonella* spp., *E. cloacae* and *Klebsiella pneumoniae*) and other species such as *Aeromonas hydrophilia* producing GES-24 enzyme [80,84].

A range of plasmid-encoded carbapenemases have been identified in bacteria of seafood origin from various countries including NDM-1 in *E. cloacae*, *E. coli*, *Vibrio alginolyticus* and *Vibrio parahaemolyticus*, NDM-5 in *E. coli*, IMI-1 in *E. cloacae*, IMI-2 in *E. aerogenes* and *E. cloacae*, KPC, OXA-48 and VIM-1 in *E. coli*, VIM-1 in *V. alginolyticus*, VIM-2 in *Pseudomonas fluorescens*, OXA-23 in *A. baumannii* [2--4, 9, 51, 85–91]. Chromosomal OXA-48-like enzymes have been reported in *Shewanella* and a new chromosomally-encoded class A carbapenemase, VCC-1, of *V. cholera* from imported retail shrimp to Canada, was recently discovered [79,92,93].

*Cross-resistance and/or co-resistance to other antimicrobial agents*. For the most part, carbapenemases display strong expanded broad-spectrum enzymatic activities for hydrolysing essentially all *β*-lactams, thus causing high-level clinically-relevant cross-resistance to carbapenems, cephalosporins of all generations and various penicillins [80,82].

Plasmidic or other mobile genetic element-associated genes encoding carbapenemases may coexist in the same multidrug resistance gene cassette regions. The latter confer co-resistance to a variety of other antimicrobials, including aminoglycosides, quinolones, amphenicols, sulphonamides and/or tetracyclines, the latter three being authorised for use in aquaculture in Canada. For instance, *bla*_NDM_-positive *E. coli* isolates of fish origin were revealed to carry the plasmid-encoded *qnrA* quinolone resistance gene [12]. Two *bla*_NDM−1_-borne IncA/C2 conjugative plasmids isolated from *V. alginoltyicus* and *V. parahaemolyticus* of different shrimp sources were found to contain Tn125 transposon and multiple genes for resistance to carbapenems, cephalosporins and penicillins (*bla*_NDM−1_), aminoglycosides (*strA*, *strB* and/or *aadA*), amphenicols (*floR*), sulphonamides (*sul1* and/or *sul2*), trimethoprim (*dfrA15* or *dfrA16*) and/or tetracycline (*tetA*) [94]. The presence of several resistance genes with carbapenemase genes highlights the potential co-selection of carbapenem resistance by structurally-unrelated antimicrobial agents.

*Transferability of resistance determinants between microorganisms*. Carbapenemase-encoding genes are often located in plasmids containing insertion sequences, transposons and/or integrons [95–97]. The capacity for horizontal gene transfer enabled by these mobile elements (via conjugation, transformation or transduction) contributes significantly to the spread of ARGs among terrestrial and aquatic microorganisms, even in distantly related bacteria, including human pathogens [95,96]. For example, conjugative transfer of carbapenemase-encoding plasmids from bacteria of seafood origin to *E. coli* has been readily demonstrated in laboratory conditions [85].

Aquatic systems such as coastal waters, lakes and rivers can act as reservoirs of AMR and facilitate resistance transmission [47,96,98,99]. In an open environmental model in the absence of antimicrobial selection pressure, Chamosa *et al*. demonstrated the transfer of *aadB* (an aminoglycoside resistance gene) and *bla*_VIM−2_ (a carbapenem resistance gene) gene cassettes into environmental bacterial strains, as well as Enterobacterales and *Vibrio* spp. [100].

#### Summary of data quality and level of concern

This section contains many different data elements, which provide a fundamental understanding of the AMR hazard and its resistance mechanism and transferability. The overall data quality score is 5.7. CREc's are expected to share similar biological features as well as resistance and transmission mechanisms and therefore information imparted in these sub-sections could be transposed to the Canadian situation. Although the publications cited are recent and peer reviewed, there is a marked lack of Canadian-specific data, particularly information concerning distribution, frequency and concentrations of the AMR hazard(s) in the food chain as well as sources and transmission routes. The consequent preponderance of data from other geographical regions and the source (reviews) are responsible, in majority, for diminishing the overall quality score. The level of concern is 3, owing to the importance of carbapenems in the human therapeutic arsenal and the paucity of Canadian data concerning the AMR hazard in salmon and shrimp.

### Information on the antimicrobial agent(s) to which resistance is expressed

#### Class of the antimicrobial agent(s)

Carbapenems are antimicrobials of the *β*-lactam class, along with penicillins, cephalosporins and monobactams, all of which are bactericidal via inhibition of cell wall synthesis. In 1985, the first carbapenem, imipenem, became available to treat complex bacterial infections in people [82]. Others soon followed, including meropenem, panipenem, biapenem, ertapenem, faropenem and doripenem [82].

#### Non-human uses of the antimicrobial agent(s) (use in aquaculture)

The use of multiple classes of antimicrobials in aquaculture is well documented [101–108]. For example, a study of four major aquacultured commodities produced in Asia demonstrated the use of aminoglycosides, antimycobacterial (rifampin), *β*-lactams (aminopenicillins, cephalosporins), phenicols, polymixins, quinolones, sulphonamides, tetracyclines and trimethoprim [108]. Although the use of carbapenems in companion animals is reported, no information concerning off-label usage in food animals could be identified in the literature [109,110]. The use of carbapenems for food-producing animals including aquaculture is not authorised in the European Union, North America and Australasia [111]. Although information on the use in some Asian and developing countries is not readily available, the cost would likely be too onerous for use in an aquaculture context. Therefore sections detailing carbapenem distribution, use and their impact on AMR as described in the Codex guidelines are not considered here.

#### Human uses of the antimicrobial agent(s)

*Spectrum of activity and indications for treatment*. Carbapenems have a broad spectrum of activity against both Gram-positive and Gram-negative aerobic and anaerobic bacteria [82]. Due to differences in activity and pharmacokinetic features of carbapenems, they are indicated for a wide range of serious bacterial infections involving the lower respiratory tract, urinary tract, intra-abdominal structures, gynaecological organs, skeletal structures, central nervous system, skin and soft tissues, heart (*S. aureus* endocarditis), as well as septicaemia [112–114].

Although carbapenems are typically reserved to treat complicated bacterial infections and not generally considered first-line treatment choices, there are exceptions [82]. For healthcare-associated complicated intra-abdominal infections, imipenem or meropenem can be a first-line empiric treatment choice in settings where there are <20% resistant *P. aeruginosa*, *Acinetobacter* or other MDR Gram-negative bacilli, where extended spectrum beta-lactamase producing Enterobacterales are present, or where >20% of *P. aeruginosa* are resistant to ceftazidime [115]. Carbapenems are also considered first-line treatment choices for empiric treatment of biliary infections in adults, be it community-acquired or healthcare-associated and in paediatric patients with complicated community-acquired extra-biliary intra-abdominal infections [116]. Likewise, ertapenem is the first-line treatment for mild to moderate infections, and imipenem or meropenem for severe infections and/or high-risk patients [115]. For invasive infections caused by *Salmonella* spp. that are resistant to ciprofloxacin and ceftriaxone, carbapenems may be the only remaining antimicrobial of choice [117]. Carbapenems are often combined with other antimicrobials to provide effective treatments in complicated infections such as those caused by MDR *Mycobacterium tuberculosis*, meningitis caused by *A. baumannii* or other ESBL-producing Gram-negative bacilli, and healthcare-associated ventriculitis and meningitis [82,118,119].

*Importance of the antimicrobial agents including consideration of critically important antimicrobial lists*. Carbapenems are a class of highly effective antimicrobials which are used for the treatment of severe or high-risk bacterial infections for which resistance development is a primary concern for human health. The WHO has classified carbapenems as ‘Critically important’ as they are the only, or one of limited available therapies to treat serious bacterial infections in people and they are used to treat infections caused by bacteria originating from non-human sources, or bacteria that may acquire ARGs from non-human sources [120]. Similarly, Health Canada also categorises carbapenems as antimicrobials of ‘Very High Importance’ and are considered essential for the treatment of serious bacterial infections and limited or no availability of alternative antimicrobials for effective treatment are available if resistance emerges [14].

In 2017, the WHO revised its Essential Medicine list, adding three new categories for antimicrobials: Key Access, Watch and Reserve. Carbapenems are in the ‘Watch’ group, due to their higher resistance potential and the recommendation that they should only be used as first or second-line treatment options for a limited number of specific indications [121]. Meropenem is also included in the ‘Key Access’ group, indicating that, in addition to the stipulations of the ‘Watch’ group, it should be widely available, affordable and quality-assured [121].

*Distribution, cost and availability*. In Canada, public funding of antimicrobials is regulated at the provincial-level, and the carbapenems registered for use in Canada are available across all provinces and territories. Meropenem, imipenem and ertapenem are the only carbapenems authorised for human use in Canada [122].

Inpatient carbapenem use is funded by all provinces, although there are some restrictions. Alberta, British Columbia and Ontario may grant special authorisation and fund carbapenems for outpatient use [123–125].

Cost per unit varies in Canada from $9.22 to $27 per 500 mg vial depending on which carbapenem is used and in which province [123,124,126].

Hospital and community pharmacy expenditure associated with carbapenem purchasing and dispensing in Canada has varied from year to year [127]. In 2010, Canadian hospitals purchased carbapenems to the value of $1279.35 Canadian dollars per 1000 inhabitant-years ($/1000 inh-yrs), and community pharmacies dispensed $33.56/1000 inh-yrs' worth of carbapenems [127]. Expenditure in 2017 for hospitals was $520.22/1000 inh-yrs, and for pharmacies $514.32/1000 inh-yrs [127]. However, use trends are not necessarily reflected by expenditure as the drivers that influence cost do not necessarily influence use.

*Availability of alternative antimicrobial agents*. Alternative treatment choices to carbapenems are limited, and most alternative treatments consist of combination therapy with a number of antimicrobial agents [115]. Combination therapy provides a significant survival benefit in CRE/CPE infections, which is even more pronounced when the combination includes a carbapenem, possibly due to a synergism between carbapenems and aminoglycosides, colistin or tigecycline [128]. Treatment choices are further complicated by the fact that optimal treatment regimes for CRE/CPE infections have not yet been established through randomised control trials, with current recommendations based on case reports, case reviews and small retrospective studies [129]. Treatment options for combination use include aminoglycosides, tigecycline, fosfomycin and rifampicin for bacteraemia and pneumonia [129]. When used in a dual antimicrobial regime, fosfomycin has a synergistic activity against most CRE/CPE, including extensively drug-resistant *K. pneumoniae*, so it may have value as a salvage treatment when treatment choices are very limited [128, 130]. Fosfomycin and rifampicin may also be used for gastrointestinal or biliary tract infections, while colistin and aminoglycosides are suitable alternatives for urinary tract infections [129]. In the case of healthcare-associated meningitis, meropenem can be substituted with aztreonam or ciprofloxacin [118]. For meningitis caused by carbapenem-resistant *Acinetobacter*, treatment options include colistimethate sodium or polymyxin B [118].

For intra-abdominal infections in paediatric patients alternative treatment choices include piperacillin-tazobactam as single agent therapy, or combinations of third or fourth generation cephalosporins, metronidazole, aminoglycosides, lincosamides and/or ampicillin [115]. In adults with complicated intra-abdominal infections, alternatives include combination therapy with fluoroquinolone, metronidazole and vancomycin [115,119].

*Trends in the use of antimicrobial agents(s) in humans*. In 2010, Canadian hospitals purchased 0.035 defined daily doses per 1000 inhabitant days (DDDs/1000 inhab-days) of carbapenems, by 2017 this has increased by almost 62%, to 0.056 DDDs/1000 inhab-days [127,131]. In 2010, this represented 3.2% of the total purchases of antimicrobials considered critically important for human medicine, by 2016, this had increased almost 41%, to 4.5%. In 2010, carbapenems represented 2.5% of the total amount of antimicrobials purchased. By 2017, this had increased by almost 50%, to 3.73%, with the largest increase from 2016 to 2017 [127,131].

Community pharmacies dispensed 17.8 DDDs/1000 inh-days of antimicrobials in 2010, with a slight 0.5% increase by 2017 [127,131]. However, the proportion of carbapenem dispensing has changed dramatically. In 2010, carbapenems accounted for only 0.006% of total antimicrobials dispensed by community pharmacies, but by 2017 carbapenems accounted for 0.094%, an increase of more than 1400%. For all antimicrobials, across hospitals and community pharmacies, the proportion of carbapenems has increased by 102% from 2010 to 2017, while the total carbapenem DDDs/1000 inhab-days have increased by 104%, from 0.036 to 0.073 [127,131]. Carbapenem use has shifted towards dispensing in communities. The reason for the shift in carbapenem use towards community dispensing is unclear.

#### Summary of data quality and level of concern

Data quality is scored as 6.9 for this section. There is current Canadian human use data allowing a higher quality evaluation. The level of concern is 3, as carbapenems are considered last-resort antimicrobials and are critically important to human medicine, and their use shows a worrying upwards trend, even if the reasons for this have not been fully elucidated. There is no documented use of carbapenems in aquaculture in Canada.

### Information on the food commodity

#### Sources (domestic and imported), production volume, distribution and per capita consumption of foods or raw material identified with the AMR hazard(s) of concern

Even though the majority of fish and seafood production in Canada is attributed to commercial fisheries, shrimp and salmon represent a relatively small percentage of the 838 550 tonnes, valued at $3.7 billion in 2018. Shrimp captures totalled only 56 948 tonnes with a value of $446 million and British Columbian commercial salmon fisheries reported captures of 10 499 tonnes (all species combined) with a value of $62 million [132]. Aquaculture in Canada accounted for a quarter of the total volume of seafood production (191 259 tonnes, valued at $1.4 billion) and 64% of this total was attributed to salmon produced in British Columbia, New Brunswick and Nova Scotia (123 184 tonnes valued at $1.1 billion) [133].

Canada imported 539 457 tonnes of fish and seafood products in 2018 with a value of $4.3 billion. The country from which Canada imported the most seafood products was the USA at 35% of the total volume [134]. This was followed by China (13%), Thailand (8%), Vietnam (7%), Peru (5%), Chile (4%) and India (4%) [134]. Salmon (60 269 tonnes) and shrimp (56 816 tonnes) are the top two imports and account for almost 11% of all seafood imported. Most shrimp and salmon consumed in Canada are farmed or aquacultured products. *Litopenaeus vannamei*, the Pacific white shrimp, is the principal farmed marine species accounting for 76% of all farmed shrimp and 45% of all shrimp from fisheries and aquaculture [135]. Giant Tiger shrimp (*Paenaeus monodon*), another marine species, and the freshwater species *Macrobrachium rosenbergii* the Giant river or Malaysian prawn are also important cultivated shrimp.

The principal farmed salmon species is Atlantic salmon (*Salmo salar*). While Canada does produce cold water shrimp from wild harvest, the main source of shrimp products eaten in Canadian households are farmed warm water shrimp imported from Thailand, Vietnam, India and China [7]. Conversely, domestic salmon consumption outweighs importations from countries including the USA, Chile and Norway [7].

In 2017, total consumption of fish and shellfish for the average person in Canada was approximately 8.71 kg per person per year [136]. From 2010 to 2017, fish consumption increased by 20%, and consumption of shellfish by 0.6%, with a total increase of seafood consumption of 16% [136]. Annual consumption of salmon and shrimp in Canada in 2017 was approximately 150 000 and 100 000 tonnes live weight, respectively [7]. More than half of the Canadian population consume seafood within any given week, including 14% consuming shrimp/prawns, 7% smoked fish, 7% raw fish, 4% scallops, 3% crab, and 2% lobster, clams, mussels and oysters. [137]. Canadian households spend 2.5% of food expenditures on fish and seafood annually, with the majority spent on salmon and shrimp [7].

*Characteristics of the food product(s) that may impact risk management (e.g. further processed, consumed cooked, pH, water activity, etc.)*. In Canada, salmon may be purchased chilled on ice, frozen, cooked, salted, smoked, cured, canned, ready-to-eat, packaged or unpackaged [138]. Shrimp are sold as whole or tails, shell-on or peeled, round or split and deveined, canned or dried. Shrimp consumption in North America is mostly raw headless, peeled or cooked shrimp, however the main retail form is frozen, heads-off, shell-on shrimp tails [139]. Normal physicochemical parameters of seafood such as pH and A_W_ are not inhibitory to *E. coli* and temperatures between 6.5°C and 49.4 °C can contribute to *E. coli* growth demonstrating the importance of the contamination of raw products in production, processing and retail activities [56].

Cooked seafood exceeds the thermal tolerance of *E. coli* and should pose no risk except for the possibility of subsequent contamination/cross-contamination. Although low levels of *E. coli* are accepted in ready-to-eat and fresh seafood according to Canadian food safety guidelines, this can be a safety concern as certain strains can cause disease at low infective doses [140,141]. *E. coli* contamination has been identified in both salmon and shrimp retail products by several authors (see Supplementary material Table S2).

*Description of the food production to consumption continuum (e.g. primary production, processing, storage, handling, distribution and consumption) and the risk factors that affect the microbiological safety of the food product of concern*. Factors contributing to microbial contamination and resistance can be found at any point along the food production to consumption continuum [142,143].

The majority of shrimp consumed in Canada are produced in South-East Asia, Central and South America. The shrimp aquaculture industry varies widely in farm types and organisation, ranging from extensive and semi-extensive growout operations with large ponds, low stocking densities and slow to non-existant water exchanges to intensive operations with small ponds or artificial structures, high stocking densities and rapid water exchanges [144]. Post-larval shrimp which are used for stocking may be wild caught or furnished by a hatchery [145]. Water sources may include tidal exchange, natural drainage and supplementation by mechanical means from natural water bodies or subsurface sources. Nutritional needs in the extensive operations are met by natural production of algae and plankton in the ponds. With intensification of production, natural feed production may be increased with the addition of organic (manuring) or chemical fertiliser and artificial feeds may be used as a supplement or as the sole ration [146]. Ponds or artificial growout units may be drained, cleaned and disinfected between shrimp crops, but this is generally limited to intensive farming operations where the size of the production unit and water flow permit [144].

Atlantic salmon are produced in Canada in the province of British Columbia, and in the provinces of Atlantic Canada. Production is divided into two major phases with egg incubation, fingerling and smolt (salmon which are physiologically adapted to salt water) production taking place in fresh water and grow out in saltwater. Broodstock can be selected from the local marine production stock, or alternatively, eggs may be purchased from national or international hatcheries. After the approximately year-long fresh water phase, from egg to smolt, the smolt are then transferred to seacages that can hold 15–30 000 market sized salmon with water quality assured by water flow [147]. All stages of salmon are fed artificial feed and biosecurity is an important aspect of production. Most if not all farms have vaccination programmes in place for common bacterial fish pathogens (e.g., *Vibrio*, *Aeromonas*). All-in all-out production for each site is the norm, although there may be some crossing between new smolts and fish waiting to be harvested within sites, and fallowing between production cycles is a common practice. Canadian Atlantic salmon are generally marketed at a size of 4–5 kg after 12–28 months at sea [148].

Antimicrobials are used in shrimp and salmon production to control bacterial disease. When necessary, they are used metaphylactically, where the entire population is treated once a certain threshold of mortality is reached. Individual treatment of shrimp or salmon is rare and antimicrobials are normally administered via medicated feed on a per weight basis [149]. Production parameters contributing to increased stress of the aquacultured species such as inadequate/substandard water quality and high stocking densities contribute to disease outbreaks which may necessitate therapeutic intervention.

In addition to AMU in shrimp and salmon production, there are several factors which can affect the selection or co-selection and mobilisation of ARGs in the aquaculture environment. These may include antimicrobial/chemical accumulation in the environment (under cages or in grow-out ponds), terrestrial contamination of water sources (sewage, agricultural runoff, manure fertilisation) and contaminated feed [150–154].

Seafood is a large component of international food trade, and often must travel long distances to arrive at the desired location. Although the activities of harvest, transport, processing and retail do not likely contribute to selection of resistant bacterial strains, these transitions are likely a key opportunity for seafood and aquaculture to be exposed to bacterial contamination [155].

Several studies have been undertaken to examine microbial contamination at different stages of the harvesting/processing and retail levels of the seafood-to-fork continuum. Uddin *et al*. [156] suggested from their study comparing bacterial flora of cultured Asian and local wild caught seafood that the normal bacterial flora is similar from both sources and the flora at the retail level likely represents a contamination from ‘repeated handling and exposure to contaminated surfaces and water during processing’ [156]. This has been echoed by other authors where harvest, transport and product manipulation have all been implicated in increased bacterial contamination [54,68,157–159].

Fish that have been heat-processed packed in sealed, chilled or frozen containers are probably least likely to expose consumers to bacteria, while those products sold fresh or frozen and require cooking pose an increased risk of exposure [140]. A certain level of bacteria is normal for seafood, especially when presented as a raw product. However, in two studies examining ready to eat shrimp, bacteria not normally associated with cooked seafood were identified. These included Enterobacterales spp., *Vibrio* spp., *Bacillus* spp. and *Staphylococcus* spp., and an *E. coli* demonstrating resistance to five classes of antimicrobials [19]. The presence of *E. coli* at the retail level indicates improper processing of ready to eat shrimp (inadequate cooking) and/or cross-contamination from employees or processing equipment, which is of concern in products consumed without further preparation to decrease bacterial presence.

Consumer behaviour, in particular unsafe food handling and preparation practices, is a critical risk factor for increasing the probability of exposure to foodborne pathogens. The most common causes of seafood-related bacterial outbreaks are improper cooking, inadequate storage, cross-contamination and use of raw ingredients in the preparation of seafood [155].

#### Summary of data quality and level of concern

The data quality score for this section is 6.2. Although recent Canadian data are available regarding domestic and imported sources, the seafood production to consumption information is mainly empirical and characteristics of the food products lack information on different forms associated with higher probabilities of foodborne infection or risk management. The level of concern is estimated at 2.5, as shrimp and salmon are commonly consumed in Canada. Although these products are not as frequently consumed as terrestrial food animals such as poultry or beef, shrimp and salmon can be consumed as cooked or raw products. Additionally, shrimp is principally an imported product and production practices and biosecurity standards may vary depending on the provenance which will affect *E. coli* contamination and dissemination.

### Information on adverse public health effects

#### Characteristics of the disease caused by the identified foodborne AMR microorganisms or by pathogens that have acquired resistance determinants via food

*Trends, prevalence and nature of AMR foodborne disease in people*^1^. *E. coli* is among the four most common foodborne bacteria causing disease in people, the others being *Campylobacter*, *Salmonella* and *Listeria*. *E. coli* are commensal Gram-negative bacilli present in the gastrointestinal tract of most warm-blooded animals, including people. Five verotypes of *E. coli*, each with distinct pathogenesis, that cause intestinal disease are recognised; enterotoxigenic *E. coli* (ETEC), enteroinvasive *E. coli* (EIEC), EHEC (enterohaemorrhagic; the most notable serotype being *E. coli* O157:H7), enteropathogenic *E. coli* (EPEC) and enteroaggregative *E. coli* (EAEC). All of these types, except for EIEC, for which no animal reservoir has ever been identified, are associated with ingestion of contaminated water and/or food [25]. *E. coli* is an important cause of extra-intestinal disease, where it is the leading cause of both community-acquired and nosocomial urinary tract infections [160,161]. *E. coli* has also been implicated in a variety of other serious conditions including intra-abdominal infections, septicaemia and systemic inflammatory response syndrome [162].

For ETEC, the infective dose is at least 10^8^ cells, although the young, the elderly and the immunocompromised are susceptible to much lower doses [163]. The infective dose for EIEC and EPEC in healthy adults is 10^6^ cells, much higher than *E. coli* O157:H7 (EHEC), the primary cause of haemorrhagic colitis which can progress to potentially fatal haemolytic uraemic syndrome, where the infective dose can be less than 100 cells [24, 164]. The CPE most frequently associated with nosocomial infections are *K. pneumoniae* and *Enterobacter* spp., whereas *E. coli* is the main cause for community-acquired CPE infection, most often urinary tract infections [165]. Spread occurs from person to person, through introduction to the body via medical devices or surgical wounds, or, in the case of intestinal disease, through contaminated food and water [166].

Although no newly emerging diseases are associated specifically with CREc, the prevalence of CRE and CPE, and the proportion of CRE and CPE that is *E. coli*, has increased in North America over the past decades. In the USA in 2013, of 140 000 hospital-acquired Enterobacterales infections, an estimated 9300 were due to CRE, with 7900 resulting from carbapenem-resistant *Klebsiella* spp. (with 520 (6.6%) attributable deaths), and 1400 due to CREc (with 90 (6.4%) attributable deaths). Almost half of patients with CRE bacteraemia die from these infections [167]. In comparison, in 2017 the estimated number of CRE hospital-acquired infections had increased to 13 100 with 1100 associated deaths [168].

The Public Health Agency of Canada (PHAC) has collected national surveillance data on CPE since 2010 from a representative sample of acute care hospitals through the Canadian Nosocomial Infection Surveillance Program (CNISP) [131]. Since 2013, the PHAC has also collected national surveillance data specific to CPE through the Canadian Public Health Laboratory Network (CPHLN), whereby provincial health laboratories voluntarily submit CPE isolates and/or aggregate data on CPE isolates [131]. Many individual provinces also have infection prevention and control surveillance and reporting protocols in place for tracking CREs in their healthcare facilities [169–171].

Since CPE was first reported in Canada in 2007, the numbers of cases have increased steadily. In a study in south central Ontario, the incidence of cases increased from 0 cases of CPE/100 000 habitants in 2007 to 0.33 cases of CPE/100 000 habitants in 2015 [165]. From 2010 to 2017 the rate of CPE infections among CNISP participating sentinel hospitals have remained low at 0.03–0.04 cases/10 000 patient-days, whereas certain CPE have rapidly disseminated to reach endemic levels in other countries [127,172–174]. While infection rates among CNISP hospitals have not significantly changed, the rate of CPE colonisation has increased more than four-fold, from 0.03 (2012) to 0.19 (2018) cases/10 000 patient-days [127,174]. This increase may be due to increased awareness, increased screening and/or increased transmission of CPE [131]. This increase is concerning as colonisation represents a reservoir of bacterial resistance.

**Fig. 1.**
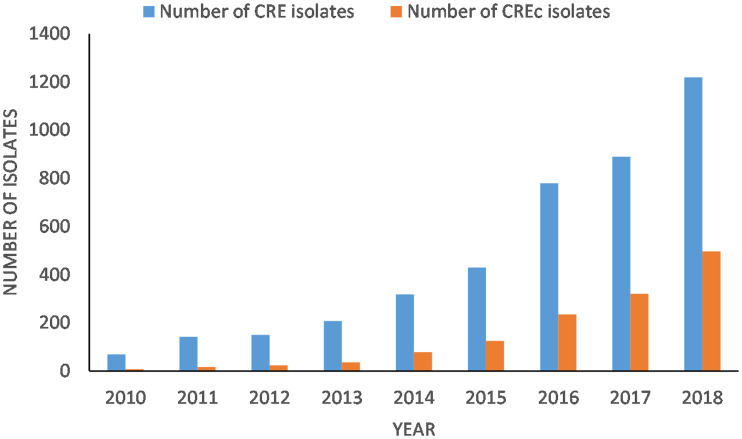
Carbapenem-resistant Enterobacterales (CRE) isolates and carbapenemase-resistant *E. coli* (CREc) reported to the Canadian Public Health Laboratory Network (CPHLN) from 2010 to 2018 (Michael Mulvey, personal communication, National Microbiology Laboratory, PHAC).

The number of CPE isolates collected by the CPHLN demonstrated an even larger increase in CPE numbers. This is likely due to increased cases of CPE in the community or among non-CNISP hospitals. The number of CPE isolates (colonisation and infection) has increased from five isolates in 2008, to 889 isolates in 2017, and to 1493 isolates in 2019, a 60% increase from 2017 to 2019 (Michael Mulvey, personal communication, National Microbiology Laboratory, PHAC). The proportion of these CPE isolates that were CPEc has steadily increased since 2010, representing almost 41% (497/1219) of all CPE isolated in 2018 (Fig. 1). Reporting of CPE to the CPHLN is voluntary and these numbers are likely to be an underestimate (Michael Mulvey, personal communication). Within the CNISP surveillance programme, *E. coli* susceptibility to meropenem has been tested since 2015, and susceptibility to imipenem and ertapenem since 2016 [175]. Overall, the proportion of *E. coli* non-susceptible (intermediate and resistant) to carbapenems has remained low varying from 0.5% to 0.8% between 2015 and 2018 depending on the carbapenem molecule examined [174].

Incidence data reported by Canadian surveillance programmes are complemented by point prevalence studies. Point prevalence surveys of Canadian acute care hospitals showed an increase in CRE prevalence, from 7% (10/143 hospitals surveyed) in 2012, to 15% (24/160) in 2016 [176,177]. The point prevalence in 2016 represented 30 patients (0.09 per 100 inpatients) that were either infected or colonised with CRE [176]. Of these, *Klebsiella* spp. were the most frequent CRE genus (34%) identified, followed by *E. coli* (28%), and *Enterobacter* spp. (22%) [176].

Enterobacterales commonly produce three types of carbapenemases, *K. pneumoniae* carbapenemases (KPC), New Delhi metallo-*β*-lactamase (NDM) and oxacillinases (OXA) [178]. From 2010 to 2014, CNISP isolated 613 CRE with 261 CPE isolates from 238 patients [97]. Out of 261 CPE isolates, 30 were *E. coli* (12%), producing KPC (*n* = 12; 40%), NDM (*n* = 10; 33%), OXA (*n* = 6; 20%) and GES (*n* = 2; 7%) [97].

**Fig. 2.**
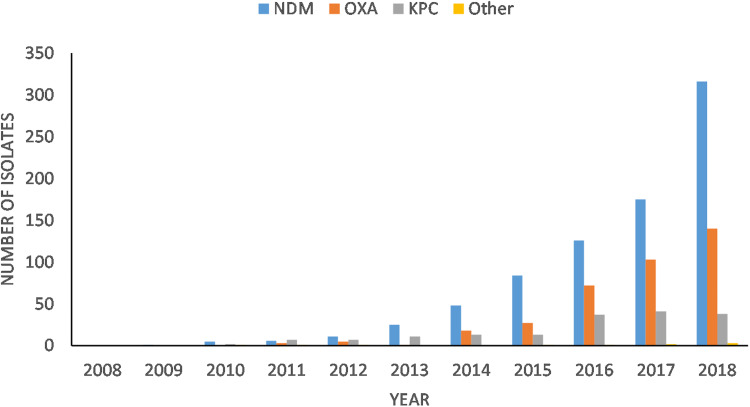
Carbapenemase types identified in *E. coli* isolates by the Canadian Public Health Laboratory Network (CPHLN) from 2008 to 2018. NDM, New Delhi metallo-*β*-lactamase; KPC, *K. pneumoniae* carbapenemases; OXA, oxacillinases (Michael Mulvey, personal communication, National Microbiology Laboratory, PHAC).

In the 2016 point prevalence study, among CRE, NDM-1 was the most prevalent carbapenemase (38%), followed by OXA-48 (24%) and KPC (14%) [176]. In Canada, during 2018, 41% of the CPE isolated produced NDM, 31.5% produced KPC and 21.4% were OXA-48-like producers (CPHLN data, Mike Mulvey personal communication). Of the 497 carbapenemase-resistant *E. coli* isolates submitted to the CPHLN in 2018, 64% produced NDM, 28% produced OXA and 8% were KPC producers, while 0.6% produced other carbapenemases (Mike Mulvey, personal communication). NDM and OXA prevalence show an increasing trend year-to-year, while *E. coli* KPC numbers have remained fairly stable (Fig. 2).

*Epidemiological pattern (outbreak, sporadic), regional, seasonal or ethnic differences in the incidence*. To understand the epidemiological pattern of CRE, including CREc, the epidemiological pattern of the resistance determinants, in particular the carbapenemases, must be understood. As the molecular resistance mechanisms of these microorganisms change, evolve and disseminate, so does the epidemiological pattern of the diseases they cause [179]. NDM producers mostly occur sporadically, except for the Indian subcontinent, the Balkan region and the Middle East, where they are considered endemic [179]. In Europe, NDM producers are commonly associated with CPE infections, while Turkey remains the epicentre of OXA-48 producers [173,179]. In the USA, NDM-, OXA-, VIM- and IMP-producing Enterobacterales are associated with sporadic outbreaks, but KPC producers are considered endemic, and the most common CRE implicated in nosocomial outbreaks and community-acquired infections [173,179–181].

In Canada, KPC-, NDM- and OXA-producing Enterobacterales are mostly associated with sporadic healthcare-associated outbreaks [127,179]. CRE implicated in HAI outbreaks in Canada include *E. cloacae*, *K. pneumonia*, *E. coli*, *Acinetobacter baumannii*, *Klebsiella oxytoca*, *Serratia marcenscens* and *Citrobacter freundii* [182–188]. In recent years, the trend of CPE in Canada has shifted from nosocomial clonal outbreaks to more cases caused by non-clonally linked Enterobacterales species [97].

Compared to poultry, meat and dairy products, foodborne diseases due to seafood consumption are generally infrequent and recent Canadian data are sparse [140]. Todd (1989) reported that in Canada in 1983, 70 foodborne outbreaks and 159 cases were associated with marine foods (fish and/or shellfish) which represented 7.3% and 2.7% of foodborne disease outbreaks and cases, respectively [189]. In a more recent publication by Todd (1997) examining seafood-associated diseases in Canada, the author notes that although information is sporadic and incomplete, infections due to various bacterial species including *Staphylococcus*, *Salmonella* and *Vibrio* among others had been reported [190]. Further, in a summary of enteric foodborne outbreaks associated with shellfish in Canada from 1998 onwards published in 2019, four of 14 outbreaks were attributed to a bacterial origin, all due to *V. parahaemolyticus* [191]. In the USA, from 1973 to 2006, 188 seafood-associated infection outbreaks (bacterial, viral and parasitic) were identified [192]. Almost half of the outbreaks (45%) were associated with molluscs, 39% with fish (21% linked to salmon) and 16% with crustaceans (50% due to shrimp) [192]. Bacteria were the aetiological agents in 76% of the outbreaks, 21% was viral and 3% parasitic [192]. Around 90% of the outbreaks associated with fish and crustaceans were bacterial, with *Vibrio* spp. the most common implicated bacteria, followed by *Clostridium botulinum*, *Salmonella* spp. and *Shigella* spp. [192]. EAEC and EHEC were each implicated in 3% of the outbreaks associated with crustaceans, but none of the fish-associated outbreaks [192]. The resistance status of these isolates was not reported. The proportion of foodborne illnesses may depend on factors such as the diet of a specific human population, as well as cooking methods employed. In Japan, for example, where fish is an important part of the diet and may be eaten raw, the proportion of outbreaks due to seafood consumption is higher. From 1973 to 1992, in the USA, Canada and the Netherland, almost 8% of the foodborne disease outbreaks were due to seafood, whereas almost 22% of the outbreaks in Japan could be linked to seafood [140].

Regional differences in KPC- and NDM-producer rates are particularly striking in Canada. KPC producers are more prevalent in central Canada compared to western Canada (1.55 *vs.* 0.24 isolates/100 000 population), whereas the trend is reversed for NDM producers (1 *vs.* 3.97 isolates/100 000) (personal communication Dr Michael Mulvey, National Microbiology Laboratory, Public Health Agency of Canada). Rates for both KPC and NDM producers have remained low in eastern Canada. Some of these differences are driven by large outbreaks at relatively few hospitals.

*Susceptible populations and risk factors*. *E. coli* is a normal inhabitant of the human and animal intestine and is commonly found in foods and the environment. However, certain pathotypes can cause severe intestinal disease. According to the CDC, populations which are more susceptible to intestinal *E. coli* infection include the young and the elderly (<5 and >65 years), those with impaired immune and health status and travellers [193]. In addition, increased risk of infections has also been associated with stress, genetic factors and the use of antimicrobials and antimotility agents [194]. Extraintestinal pathogenic *E. coli* (EXPEC) is the most common Gram-negative bacterial pathogen in humans and is an important cause of urinary tract infections, bacteraemia and meningitis [195]. There is an increasing body of evidence demonstrating that food is an important source of *E. coli* causing extraintestinal infections in humans [196]. Risk factors for *E. coli* urinary tract infections include female gender, men aged >45 years with prostatic hypertrophy, urinary catheterisation, mechanical manipulation, obstruction and diabetes mellitus [197]. These factors are of particular importance as the urinary tract is the most common source of bacteraemia in adults [195].

Some of the most prominent host risk factors for CRE acquisition include prior stay or admission from a long-term care facility, poor functional status, intensive care unit admission, AMU (carbapenems, cephalosporins, fluoroquinolones), multiple trauma, mechanical ventilation, serious comorbid conditions, solid organ or stem-cell transplantation and indwelling urethral or central venous catheters [198–204]. These risk factors are more prevalent with carbapenem-resistant *K. pneumoniae*, which is more associated with nosocomial infections than *E. coli* [165].

In Canada, colonisation or infection by CRE, and especially *E. coli*, has been associated with medical tourism, a history of travel to high-risk countries and/or healthcare encounters abroad [165, 187]. A public health report from Ontario noted that the top three risk factors for CRE cases include chronic illness/underlying conditions, inpatient hospitalisation and travel outside of Canada [205]. Infections due to CRE, including CREc, are more associated with sporadic occurrences and outbreaks within healthcare settings, rather than contaminated food consumption [206]. One of the first Canadian cases of CREc infection linked to international travel and hospitalisation occurred in 2010 involving a traveller to India [207]. Since then, multiple travel-related cases have been identified [184,185,188]. From 2010 to 2013, 12 patients infected or colonised with CRE with histories of recent international travel and hospitalisation while abroad were identified in Alberta [208]. Four out of 17 (24%) CPE identified were *E. coli*, and all of them were associated with travel to India [208]. It is believed that the combination of nosocomial transmission of CPE and international healthcare encounters are the main drivers of its persistence within Canada [97].

*Regional, seasonal and ethnic differences in the incidence of foodborne disease due to the AMR hazard*. No ethnic predilection and no regional or seasonal differences were noted in the literature concerning disease caused by *E. coli* (resistant or non-resistant bacteria) of seafood origin. However, some regional and seasonal trends in seafood-associated bacterial outbreaks from 1973 to 2006 have been noted in the USA, where a higher percentage of outbreaks occurred in coastal states, and typically peaked during the late summer months [192]. This could be a reflection of a combination of warmer seawater temperatures which are permissive for bacterial pathogen growth and seafood consumption patterns.

Despite the fact that CREc has been found in various places across the globe, no large-scale outbreak of CREc from any source has been identified. However, if carbapenem resistance genes are successfully transferred to an *E. coli* strain capable of rapid dissemination, the results could be overwhelming [200].

*Consequences of AMR on the outcome of the disease (burden of illness (BOI))*. The measure of the consequences of disease is often described as the BOI which can describe human health, social aspects and costs to society associated with the disease in question. AMR in bacteria causing infections in humans is associated with an increase in the severity of such infections and a higher probability of treatment failure, leading to longer durations of infections, increased frequency of bacteraemia, increased and prolonged hospitalisation, as well as increased risk of mortality [209]. These infections also have an economic impact as these patients often require prolonged courses of more expensive alternative antimicrobial therapies, extraordinary diagnostic and infection control efforts, and loss of productivity [210,211].

Infections caused by CRE tend to be invasive and due to the MDR nature of many CRE isolates, therapeutic options can be severely limited, resulting in adverse clinical outcomes [176,210,212]. Bacteraemia due to CRE, compared to bacteraemia due to carbapenem-susceptible Enterobacterales, is associated with a higher probability for bacteraemia persistence, and recurrence [213]. One of the factors that may contribute to the poor outcomes of carbapenem-resistant infections is the failure to provide timely effective antimicrobial therapy, which may be due to a delay in diagnosis, lack of effective alternative therapies, low sensitivity of automated screening tests, slow bacterial culturing methods and/or a lack of awareness of CRE by physicians and laboratory technicians [212,214–216].

No national BOI data specifically associated with CRE or CREc, such as the number of cases or incidence and outcomes of treatment failures, are routinely collected in Canada. In the USA, incidence of CRE infection is estimated to be 2.93 cases per 100 000 persons, which was extrapolated to 9418 CRE infections in 2015 at the national level [217]. With this incidence rate and assuming 26% attributable mortality, it is estimated that CRE infections in the USA are attributable to 840 deaths, 8841 quality-adjusted life-years lost and over $275 million in hospital costs per year [217]. While clinical outcomes and risk factors of carbapenem-resistant *K. pneumoniae* have been well documented, far less is known about CREc infections [202–204,218].

Factors that can influence mortality rates include the type and site of infection (e.g. meningitis, bacteraemia or urinary tract infection), co-morbid conditions, prior AMU and length of hospital admission pre-infection. In a systematic review undertaken by MacKinnon *et al*. [219] examining the health and healthcare burden due to antimicrobial-resistant *E. coli* in humans found resistant *E. coli* infections were associated with significant 30-day and all-cause mortality burden [219]. Published attributable mortality rates for CRE ranged from 26% to 58%, with a similar range of 18–65% for CPE [203,220,221]. The median duration of hospitalisation was 19 days for CRE, and 29 days for CPE [212,221]. In Canada, the all-cause mortality rate for CPE, from 2010 to 2017, was 17–18% [97,127].

A systematic review that examined deaths attributable to CRE concluded that patients with bacteraemia due to CRE are two times more likely to die than those with bacteraemia due to carbapenem-susceptible Enterobacterales, and that carbapenem resistance among Enterobacterales was independently associated with higher mortality rates [203]. A US study modelling CRE infection outcome found that the cost due to CRE infection was greater than that associated with chronic disease or some acute diseases annually [217]. Two studies compared outcomes of CREc and carbapenem-susceptible *E. coli* infections. The first recorded that those patients with CREc had worse disease severity, longer hospitalisation periods and higher in-hospital mortality rates, and similarly, the second indicated that total diagnostic, treatment cost and mortality are significantly higher in CREc as compared with susceptible cases [222,223].

#### Summary of data quality and level of concern

The overall average data quality for this section is 6.4. This is largely due to the lack of Canadian BOI data specific to CREc constituting a major data gap. However, given the available information on CRE in general, it is reasonable to expect similar increases in morbidity and mortality, as well as loss of treatment options and treatment failures from CREc, compared to infections caused by susceptible bacteria. The level of concern is estimated at 3, based on the availability of sufficient information to confirm CRE's association with worse disease outcomes.

### Risk management information

#### Identification of risk management options to control the AMR hazard along the production to consumption continuum

The risk related to the presence of foodborne-resistant microorganisms in seafood is multi-faceted and complex. Mitigating measures targeting only one aspect of the food to fork continuum may be helpful in decreasing risk, but if other elements are ignored, the gains in one sector may be negated in another. Therefore, in as much as possible, a comprehensive and multi-pronged approach to risk reduction should be advocated.

*Measures to reduce the risk related to the selection and dissemination of foodborne AMR microorganisms*. Reduction of the use of antimicrobials in aquaculture production is an important recommendation to decrease selection, co-selection and mobilisation of ARGs in the production of aquatic food animals [209]. A strong correlation has been demonstrated between contamination of the aquatic environment with various antimicrobials and the occurrence of MDR bacteria, even when the contaminant concentration is low [224]. Recommendations for prudent and responsible use of veterinary medicines in aquaculture have been recently published by the FAO and could/should be adapted to regional and national realities [225]. Adequate support for an aquaculture industry is vital, including extension services, support and availability of appropriate therapeutic interventions and diagnostic services (veterinarian and biologist expertise), as well as the establishment and enforcement of regulations concerning AMU [226]. This type of environment may be present in more developed countries but remains a challenge in many of the major aquaculture-producing regions of the world. Although the beta-lactam class of antimicrobials is not used in Canadian aquaculture, decreasing overall AMU is considered an important recommendation in order to limit co-selection and mobilisation of ARGs.

Several health management alternatives to antimicrobials can be utilised to decrease AMU dependence. Vaccination has been used to great effect in the Norwegian salmon industry where AMU has decreased more than 99% following the institution of oil-adjuvanted vaccines to control bacterial disease [227–229]. Similar progress has been reported in British Columbia [106]. However, vaccine development is a long and expensive process and the number of different species currently cultivated in the aquaculture setting is daunting. Additionally, vaccination is not a current option in animals with more primitive immune systems such as the crustaceans [230]. Optimising healthcare management and culture practices takes on an even greater importance in these instances. The use of appropriate culture practices and conditions for the aquatic species in question is paramount. Attention to water quality (e.g. temperature, oxygen), stocking densities, nutrition, biosecurity and the use of disease-resistant/disease-free (specific pathogen free) stock, among others, play an important role in the health and capacity to resist the disease of aquatic organisms and consequently help to reduce the number of therapeutic interventions required [106,108,231].

All antimicrobials used in the Canadian aquaculture industry are approved by Health Canada, and both freshwater and marine aquaculture facilities are required to report AMU under the Aquaculture Activities Regulations administered by Fisheries and Oceans Canada [232]. Regulatory environments differ from country to country, and seafood is imported into Canada from countries which permit the use of several classes of antimicrobials including beta-lactams. The Safe Food for Canadians Regulations require the preparation of a preventive control plan for seafood importations which identify hazards and control measures put into place to ensure a safe food product. These are based on the Codex Alimentarius General Principles of Food Hygiene CAC/RCP 1–1 969, and address food hazards by prevention, elimination or reduction to an accepted level [233]. Audits of seafood suppliers by the importer or a competent third party are used to identify hazards and corrective actions. Alternatively, importation from an authorised country which is overseen by an inspection system approved by the Canadian Food Inspection Agency (CFIA), providing the same level of protection as Canadian systems is possible [234]. This is currently a requirement for all shellfish importers [234]. Random sampling of imported seafood is undertaken by the CFIA, with an emphasis on first-time importers and those with a history of non-compliance with Canadian standards. Acceptable bacterial levels in sampled seafood, including *E. coli*, are outlined in the CFIA guidelines [141]. Regulations also require testing for chemical residues, including antimicrobials, in imported and domestic seafood ensuring compliance with minimum residue limits (MRLs) [235]. Although established MRL surveillance can help maintain desired antimicrobial residues in relation to aquacultured products, MRLs alone do not address the risk of AMR. Additionally, current microbiological testing is designed to ensure innocuity and does not include an evaluation of AMR. Surveillance using modern molecular techniques would be useful in detecting microbiological hazards including AMR genes of concern.

Site selection for the aquaculture operation is crucial for the health of the cultured organisms as mentioned previously. Contamination of the aquatic environment from anthropogenic/terrestrial sources, such as sewage and agricultural runoff, by antimicrobials, ARGs and pathogenic bacteria has been reported by several authors [151,153,236–238]. In Canada, for example, siting requirements and permits are governed by federal and/or provincial regulations to prevent this occurrence. The use of organic fertilisation or ‘manuring’ is not used in salmon production, but has been reported in shrimp culture conducted by small-scale or family farming in developing countries and is discouraged when aquaculture products are destined for exportation due to quality issues [144,239].

Interventions at the processing and retail levels are equally important. Some post-harvest critical control points for control of pathogens in shrimp and salmon include chilling immediately in an ice-water slurry at the harvest site, proper cooking, rapid chilling after cooking and frozen storage [56,240]. Although rapid cooling and maintenance of the cold chain does not necessarily eliminate pathogenic bacteria, decreasing the duration of exposure to ambient temperatures and the rapidity of cooling/freezing following harvest is critical to keeping microbial counts low and ensuring seafood safety/quality. Bacterial populations including possible human pathogens have been shown to increase more quickly in seafood stored at temperatures above 0 °C [241]. *E. coli* populations specifically increase in seafood exposed to higher storage temperatures [242]. The freezing of seafood does not eliminate bacteria and should only be considered as a means to preserve seafood and prevent bacterial multiplication. Cooking shrimp and salmon to an internal temperature of 145 °F (63 °C) kills bacterial pathogens such as *E. coli*, so safety concerns are normally focused around fish being improperly cooked or consumed raw [140].

*Measures to minimise the contamination and cross-contamination of food by AMR microorganisms*. Hazard Analysis and Critical Control Points (HACCP) or quality assurance programmes have been developed for aquaculture production and processing and are generally used for higher value products such as salmonids, shrimp, shellfish and catfish or where compliance with sanitary requirements is required for importing countries [243,244]. Since 1997 in the USA, all seafood processors are required to implement a HACCP programme to their operations in the country and foreign countries that export seafood products to the USA [245]. The Safe Food for Canadians Regulations require that seafood importers prepare, keep, maintain and implement a written preventive control plan to demonstrate how hazards and risks to food are addressed to obtain an import licence. Further, a risk base approach to inspection and sampling is undertaken to ensure importer compliance [246]. However, inspections target pathogen presence and chemical residues (including antimicrobials) but not AMR [141,235]. The requirement of a HACCP or other quality control process programme at the processing and retail levels can help avoid contamination and cross-contamination issues. The establishment of quality assurance programmes does not guarantee a safe seafood product for consumers, however they do provide a regulatory framework from which risk-based sampling can be undertaken. Within this framework, various methods are used to avoid contamination issues in processing waters, processing surfaces and seafood products thus limiting AMR hazards. Shrimp farmers prefer to sell fresh shrimp because it minimises their need for processing permits, and decreases the requirement for formal HACCP food safety programmes [247].

Thermal inactivation (or cooking) is an efficacious method for controlling bacterial contamination and/or proliferation which can be used at processing, retail and consumer levels. At the processing level, a comparison of raw block frozen shrimp and cooked individual quick freezer shrimp revealed that the cooked product consistently demonstrated the lowest density of total aerobic bacteria [248,249]. These findings were echoed in another study where *E. coli* was absent from cooked shrimp in the processing facility examined as compared to raw products [20]. At the household level, cooking has been shown to be efficacious in drastically diminishing faecal coliform counts in seafood [158]. However, as effective as thermal inactivation has proven to be, cross-contamination following cooking may render the microbial gain moot.

Seafood products for which bacterial contamination has been prevented and/or has been subjected to processing conditions that kill bacteria or prevent their growth are less likely to act as vehicles for AMR [140]. Such processes include salting, marinating, fermenting and hot or cold-smoking, among others.

Several studies demonstrated that rinsing seafood with chlorinated water at different stages of processing is beneficial in reducing bacterial contamination. In a study of Indian prawn processing units, bacterial contamination was low at harvest and increased after transport and receiving at the processing unit. Subsequent washing of shrimp with chlorinated water reduced significantly the faecal coliforms present [66,158,250].

Ozone and electrolysed oxidising water have been shown to be effective in decreasing bacterial counts and slowing bacterial proliferation in seafood. Minimal ozone treatments to Pacific white shrimp and other seafood species have been noted to decrease significantly the total viable count between ozone-treated and control samples [61,251,252]. In addition to reducing *E. coli* and other pathogens on fish and in shellfish, electrolysed oxidising water may act as a sanitising solution for working surfaces as well [253,254].

Processing water has been identified as a source of bacterial contamination in seafood processing plants. UV treatments can be efficacious in inactivating *E. coli* in a shrimp processing plant environment. In a study examining *E. coli* in shellfish processing water, total inactivation of *E. coli* was reported after 15 s of treatment at optimal operating conditions [255].

Irradiation of foodstuffs had been shown to be an effective method of food preservation in several countries. It can be utilised to prolong shelf-life by reducing bacterial loads responsible for spoilage and decrease the presence of bacteria including pathogenic species [256–262]. Although irradiation of shellfish is allowed in the USA and other countries, it is not currently permitted in Canada [263].

#### Effectiveness of current management practices in place based on surveillance data or other sources of information

Several management practices are currently used or recommended in the aquaculture industry to decrease AMU and microbial contamination in the seafood to fork continuum. Though not an exhaustive list, many were examined in the previous section. Although surveillance programmes exist for terrestrial species in North America and Europe, aquacultured species are not currently sampled. This major data gap renders evaluation of the efficacy of management practices difficult.

#### Summary data quality and level of concern

At the present time, it is not possible to evaluate the effects of management changes on AMU/AMR in the aquaculture setting and upon the seafood to fork continuum. The absence of targeted surveillance programmes capable of following the prevalence of carbapenem-resistant organisms in salmon and shrimp and permit the evaluation of outcomes of risk management decisions constitutes a significant data gap.

### Evaluation of available information and major knowledge gaps

For the purpose of this risk profile, where appropriate, each section was summarised qualitatively, highlighting uncertainty of information and data gaps (Supplementary material SE1). The most important data gap identified is the lack of AMR surveillance data targeting domestic and imported seafood. Several point prevalence studies were identified in the literature from various countries. However, the lack of information concerning pathogen prevalence in seafood types of concern, salmon and shrimp in the Canadian context, was flagrant. Additionally, prevalence studies lend themselves poorly to trend analysis and evaluation of the efficacy of interventions on the presence of AMR in seafood. Without a baseline provided by surveillance, the potential risk of carbapenem-resistant *E. coli* in retail salmon or shrimp may be under or overestimated.

## Discussion

The development of a risk profile as described by the Codex Guidelines for Risk Analysis of Foodborne AMR is among the preliminary foodborne AMR risk management activities, once an AMR food safety issue has been identified. The risk profile describes and defines the food/bacteria/antimicrobial combination. At its culmination, it will guide decision makers towards next steps in the risk analysis process which includes the following: no further action is needed, the need and mechanism to obtain additional information to fill data gaps, the implementation of risk mitigating measures for identified risks or the commissioning of a foodborne AMR risk assessment [1]. The principle factors, which motivated interest in this risk profile, were the identification of CRE in Enterobacterales in targeted Canadian retail seafood sampling and an increasing prevalence of CRE in the human population, endangering the efficacy of carbapenem antimicrobials.

Worldwide seafood production is expected to grow over the next decade. It is estimated that by 2030, 62% of food fish will come from aquaculture (109 million tons), an increase of 26 million tons over 2018 [264,265]. Shrimp and salmon production is predicted to increase by 9% and 4%, respectively [266]. Retail shrimp and salmon are primarily aquacultured products; salmon produced domestically and shrimp imported in Canada. As such, they are more likely to be exposed to antimicrobials than wild caught seafood. Although carbapenem use in aquaculture has not been reported or expected, it has been shown that multiple classes of antimicrobials are currently utilised globally, and co-selection of ARGs is an important consideration [98,108]. Canadian seafood consumption is also projected to grow by up to 9% in the next decade, an important consideration for potential increased human exposure to microbiological hazards, if present. [7].

The number of CPE isolates submitted to Canadian provincial public health laboratories has increased (from 779 in 2016 to 1493 in 2019), as well as the prevalence of CRE/CPE, and the proportion of CRE and CPE that is *E. coli* [127]. Additionally, the dispensing of carbapenems by human hospital and community pharmacies has increased by 102% from 2010 to 2017 as a proportion of total of all dispensed antimicrobials [127,131]. Although CREc infections in people are most commonly associated with chronic disease/hospitalisation and medical tourism and travel, in Canada, rather than foodborne sources, the recent identification of carbapenem ARGs in Canadian retail seafood could potentially indicate a domestic source outside of the healthcare system or travel-related transmission. The lack of AMR surveillance in retail seafood makes contribution from this source difficult to estimate.

Similarly to the findings described by Carson *et al*. [8], the risk profile outline provided by Codex proved to be a useful tool for the development of this document [8]. Carson *et al*. [8] noted similar concerns including the duplicative nature of some of the recommended elements and the resource-intensive nature of the process. However, the duplicative nature also ensures that critical information is captured and the iterative aspect of the suggested elements of the Codex Guidelines provides the necessary flexibility to address different aspects of AMR food safety issues or hazards. The outline was particularly useful when describing existing data gaps.

Several data gaps were highlighted by this risk profile. The principle gap was the lack of Canadian information concerning distribution, frequency and concentrations of the AMR hazard in these food animal species/food, which is necessary to evaluate trends, guide antimicrobial stewardship initiatives or risk management options. Information concerning the BOI associated with CREc is not collected or analysed routinely in Canada, and few studies were identified in the literature which evaluated the BOI of CREc in humans. These types of data are important in risk analysis for hazard identification, and for comparisons of the BOI before and after interventions helping guide future risk management interventions. The proportion of Canadian resources cited in this risk profile was small, with the exception of the active surveillance programmes in Canadian hospitals, which provided valuable and recent human prevalence data, as well as seafood importation, domestic seafood production and consumption data, which were well documented. Much of the data described in this risk file originated from other geographical regions. This is to be expected, as with studies examining the shrimp processing chain, for example, where shrimp are grown and processed in southeast Asia and are imported and distributed in Canada. Though these sources are helpful, they provide little insight into the Canadian production, distribution and retail context.

The information presented in this risk profile indicates that seafood can be contaminated with CREc and CPE and have the potential to act as a reservoir for bacteria and their ARGs. The findings permit the definition of CREc of shrimp and salmon available for purchase by consumers in Canada as an AMR food safety issue. According to the Codex Guidelines for Risk Analysis of Foodborne AMR, the information generated here could be used to make provisional decisions concerning risk management options, and providing advice as to whether a risk assessment is needed. For example, targeted testing of AMR in imported shrimp could be used to obtain more information to further define the AMR food safety issue and address one of the key data gaps. A qualitative or quantitative foodborne AMR risk assessment could also be considered to characterise the magnitude of the risk posed by this issue and evaluate potential risk management interventions. Three approaches are described by FAO/WHO for the risk assessment of a microbiological hazard in food including, estimating an unrestricted or baseline risk, comparing risk intervention strategies and a research-related study or model [267]. The unrestricted or baseline risk approach is cited as being most often used in import-risk analysis, where for example, information concerning risks associated with production, transport and processing before reception at the importing countries borders is poorly understood or unknown [267]. The Codex Guidelines for Risk Analysis of Foodborne Antimicrobial Resistance describe four components of a risk assessment including hazard identification, exposure assessment, hazard characterisation and risk characterisation [1]. The hazard and the AMR food safety issue were well described in the current risk profile, which borders on meeting the requirements for a qualitative risk assessment; however, the lack of baseline prevalence data in Canadian retail seafood results in an inability to create a useful quantitative risk assessment model at this point in time. An exposure assessment will require information concerning transmission and exposure pathways, AMU in the different phases of production and frequency and concentrations of the AMR hazard from harvest to retail [1]. The use of AMR surveillance in seafood would help fill some of the identified data gaps, particularly for the exposure assessment. Additionally, surveillance data would aid in the understanding of the prevalence of AMR microorganisms in seafood, the identification of trends and in the evaluation of the consequences of risk management interventions through future risk profile or risk assessment activities.

The Canadian regulatory framework already in place could be leveraged as a risk management and information gathering tool. The regulations governing AMU in Canadian aquaculture are well developed; however, the AMU regulatory environment in importing countries may be unknown or different from the Canadian situation. It has been suggested that lack of appropriate regulatory structures and enforcement in other countries contributes to inappropriate AMU in the seafood and aquaculture industries and results in the selection and spread of AMR among bacteria found in fish and shellfish, aquaculture environments, animals and humans [149,226,268]. The regulatory divergence which may be present between domestic and imported aquaculture products is important and a thorough examination of the risk associated with the regulatory environment and microbiological hazards in imported seafood has yet to be undertaken. As noted previously in this risk profile, in Canada, seafood importers are required under the Safe Food for Canadians Regulations to establish a preventive control plan to minimise health risks associated with seafood exposure. Canadian quality control programmes also monitor seafood for drug and chemical residues as well as bacterial contamination, but not AMR. European AMR surveillance programmes have recognised the importance of monitoring both domestic and imported food products to understand global AMR impacts [269]. In Europe, imported meats including poultry, beef and pork are sampled to monitor AMR in *E. coli* and *Salmonella*, including resistance to carbapenems [269]. The establishment of AMR surveillance of imported and domestic seafood in parallel with the existing regulatory requirements would help fill the most prominent data gaps.

To investigate the interrelationship between aquaculture, the environment and human health, a holistic or One-Health approach is needed. This would be facilitated in production environments where all elements of the chain of production are available for analysis, such as in the case of domestic production. Although the interconnection between terrestrial and aquatic ecosystems is complex, new technologies and increasing access to genetic tools such as whole genome sequencing can facilitate understanding these relationships. Whole genome sequencing could be used in conjunction with established monitoring activities, as a surveillance-based risk management tool, to provide insight concerning the characteristics of AMR microorganisms and determinants, the transfer and dissemination of genetic elements in the aquaculture seafood to fork continuum, and the links between resistance and virulence and fitness traits.

The absence of CREc in seafood examined in Canada to date is reassuring, however, the ARGs identified in Enterobacterales isolated from retail seafood speaks to their presence. In order to address the principle data gap, AMR surveillance needs to be undertaken to evaluate prevalence and evolution of CREs in seafood. Salmon and shrimp, being the two most important seafood products consumed in Canada and representing domestic and imported seafood products, would be appropriate initial targets for surveillance activities. The incorporation of whole genome sequencing into surveillance activities, either with a species-specific or a metagenomic approach would be a valuable addition to understanding the possible risk from this AMR food safety issue.

The objectives of this paper were to evaluate the AMR food safety issue represented by carbapenem-resistant *E. coli* originating from salmon and shrimp available for purchase by consumers in Canada, utilising the Codex Guidelines. This was our first experience applying the Guidelines to non-terrestrial food animal species. They provided a transparent and structured format for inclusion of the additional considerations of the water environment. The Guidelines were also able to accommodate a complex issue regarding multiple food products/food animal species with both domestic and international considerations. The experience gained in the production of this and previous risk profiles will improve the rapidity and efficiency of future risk profiles, where common commodity and hazard themes will permit referencing or updating previously published material.

## Data Availability

All data generated or analysed during this study are included in this published article (and its Supplementary information files) except Figures 1 and 2, as detailed in the text.

## Footnotes

1 The recommended Codex risk profile elements of ‘Trends in AMR foodborne disease’ and ‘Frequency and severity of effects of disease’ have been combined in this manuscript.

## Supplementary material

For supplementary material accompanying this paper visit http://doi.org/10.1017/S0950268822001030. click here to view supplementary material
