# Supplementary material for: Carbapenem-resistant Escherichia coli from shrimp and salmon available for purchase by consumers in Canada: a risk profile using the Codex framework
Source: Epidemiol Infect. 2022 Jun 29;150:e148. doi: 10.1017/S0950268822001030 (PMC9386791; doi:10.1017/S0950268822001030)
Supplement: Supplementary file 1 [file hygsup.zip › S0950268822001030sup001.docx]

# Epidemiology and Infection

**Carbapenem-resistant *Escherichia coli* of shrimp and salmon origin - A risk profile using the Codex framework**

D. Loest^1^, F. C. Uhland^1*^, K. M. Young^1^, X-Z. Li^2^, M. R. Mulvey^3^, R. Reid-Smith^1^, L. M. Sherk^1^, C. A . Carson^1^

**^1^**Centre for Food-borne, Environmental and Zoonotic Infectious Diseases, Public Health Agency of Canada, Guelph, Ontario, Canada,

**^2^**Veterinary Drugs Directorate, Health Products and Food Branch, Health Canada, Ottawa, Ontario, Canada

^3^National Microbiology Laboratory, Public Health Agency of Canada, Guelph, Ontario, Canada

Corresponding author

*E-mail: [frederick.uhland@canada.ca](mailto:frederick.uhland@canada.ca)

**Supplementary Material**

## Supplementary Table S1: Summary of data quality, level of concern, data gaps

| **Sections** | | **Data available (Y/N/S/L^#^)** | **New Data Needed to Make Risk Management Decision?** |
| --- | --- | --- | --- |
| 1. Description of the AMR food safety issue (per Codex definition) | | N/A | |
| 2. Information on the AMR organism /determinant | | Data Quality Score summary: 5.7; Level of Concern summary: 3 | |
| 2.1. Characteristics of carbapenem resistant *E. coli* (CREc) | 2.1.1. Sources and transmission routes | N | Yes  Surveillance of retail seafood in Canada, limited to nil. |
|  | 2.1.2. Pathogenicity, virulence, and linkages to resistance of particular strains | S | Yes  More data are needed on the genetic linkages of pathogenicity, resistance, and virulence characteristics, and its impacts on human illness. Information from whole genome sequencing (WGS) would be beneficial. |
|  | 2.1.3. Growth and survivability, including inactivation in foods (D-value, minimum pH for growth) of *E. coli* in seafood to fork continuum | Y | No  Guidelines for bacterial growth and survivability in seafood are published. |
|  | 2.1.4. Distribution, frequency and concentrations in the food chain | L | Yes  Yes, more data needed on *E. coli* and resistance prevalence/concentrations within shrimp and salmon products imported and produced in Canada to quantify the risk posed by the products to Canadians. |
| 2.2. Characteristics of carbapenem resistance in *E. coli* isolated from retail shrimp and salmon | 2.2.1. Resistance mechanisms and location of the resistance determinants | Y | No  Carbapenem resistance and their mobile genes are well characterized. |
|  | 2.2.2. Cross-resistance and/or co-resistance to other antimicrobial agents | Y | Yes  Even though phenotypic data exist in Canada, knowledge of the genes involved in cross-resistance/co-selection would assist decision making about interventions. Information from WGS would be beneficial |
|  | 2.2.3. Transferability of resistance determinants between microorganisms | Y | Yes  Antimicrobial resistance gene (ARG) transfer has been proven, however more information could be gathered about the rate of transfer. WGS would be beneficial in investigating this aspect |
| 3. Information on the antimicrobial agent(s) to which resistance is expressed - carbapenems | | Data Quality Score summary: 6.9; Level of Concern summary: 3 | |
| 3.1. Class of the antimicrobial agent(s) | | Y | No |
| 3.2. Non-human uses of carbapanems | Carbapenems are not used in aquaculture | N/A | Yes. Continued surveillance of the literature should be undertaken to identify changes in usage practices |
| 3.3 Human uses of carbapenems | 3.3.1 Spectrum of activity and indications for treatment | Y | No |
|  | 3.3.2 Importance of the antimicrobial agent, including consideration of critically important antimicrobial lists | Y | No |
|  | 3.3.3 Distribution, cost and availability | Y | No |
|  | 3.3.4 Availability of alternative antimicrobial agent(s) | Y | No |
|  | 3.3.5 Trends in the use of the antimicrobial agent(s) in humans and information on emerging diseases due to microorganism(s) resistant to the antimicrobial agent(s) or classes. | L | Specific quantitative data regarding disease manifestations related to CREc in humans are needed |
| 4. Information on the Food Commodity – salmon and shrimp | | Data Quality Score summary: 6.2; Level of Concern summary: 2.5 | |
| 4.1 Source(s) (domestic or imported), production volume, distribution and per capita consumption of foods or raw materials identified with the AMR hazard(s) of concern. | | Y | Yearly Canadian production data are available. Surveys of per capita consumption are repeated regularly (e.g. Foodbook 2.0) |
|  | 4.1.1 Characteristics of the food product(s) that may impact risk management (e.g., further processed, consumed cooked, pH, water activity, etc.) | Y | Unlike other types of animals products (e.g., poultry or beef), seafood may consumed raw (e.g., Sushi), making growing, processing and retail contamination concerning. Lacking information on different forms associated with higher probabilities of foodborne infection, or impact risk management. |
|  | 4.1.2 Description of the food production to consumption continuum (e.g., primary production, processing, storage, handling, distribution and consumption) and the risk factors that affect the microbiological safety of the food product of concern. | Y | Yes  The majority of the shrimp consumed in Canada are imported. Regulation differences concerning AMU, hygiene etc. need to be considered in any analysis of risk. There are many opportunities throughout the food production to consumption continuum for contamination to occur. Would benefit from Canadian-specific quantitative studies. |
| 5. Information on adverse public health effects | | Data Quality Score: 6.4; Level of Concern: 3 | |
| 5.1 Characteristics of the disease caused by the identified foodborne AMR microorganisms or by pathogens that have acquired resistance determinants via food | 5.1.1. Trends prevalence and nature of AMR foodborne disease in people | Y | Yes  Surveillance of CRE infections in Canada is currently undertaken by the Canadian Nosocomial Infection Surveillance Program (CNISP) and the Canadian Public Health Laboratory Network (CPHLN). Trends are increasing; Up to date Canadian passive surveillance data are available but there are data gaps with regards to the rate and prevalence of CREc, and on the burden of illness caused by CREc. However, cases attributed to food consumption are unknown. WGS analysis could provide information concerning source attribution of CREc isolates. |
|  | 5.1.2. Epidemiological pattern (outbreak or sporadic) regional, seasonal or ethnic differences in the incidence | S | Yes  Epidemiological patterns thus far identified are associated primarily with hospital care, where surveillance activities are concentrated. Surveillance of food animals has been undertaken by CIPARS but salmon, shrimp and seafood are not routinely included. Global dissemination of CPE is of concern. |
|  | 5.1.3. Susceptible population and risk factors | L | Yes  Information concerning risk factors and susceptible populations is unknown. Consideration of populations at risk, which may consume seafood raw. |
|  | 5.1.4. Regional, seasonal and ethnic differences in the incidence of foodborne disease due to AMR hazard(s) | S | Yes  Information concerning risk factors and susceptible populations is unknown. Consider here ethnic populations which consume seafood raw. Travel associated CRE infection has also been demonstrated. |
|  | 5.1.5. Consequences of AMR on the outcome of the disease Burden of illness (BOI) | N | Yes  Information examining BOI of CREc infections is limited. CRE's are associated with worse disease outcomes |
| 6. Risk management options | | Data Quality Score: 5.6; Level of Concern: 3 | |
| 6.1. Identification of risk management options to control the AMR hazard along the production to consumption continuum both in the pre-harvest and post-harvest stages | 6.1.1. Measures to reduce the risk related to the selection and dissemination of foodborne AMR microorganisms(s) | L | Farm level data is difficult to obtain from importing countries, and instigating change from importers of seafood to Canada would be difficult. Risk reduction in imported seafood products would likely target processing and retail sectors where governmental and hygienic controls (HAACP) are in place. |
|  | 6.1.2. Measures to minimize the contamination and cross-contamination of food by AMR microorganism(s) | S | Several studies have demonstrated the utility of HAACP programs in seafood processing plants. A qualitative description of what might be risk management options is available; quantitative data supporting the effectiveness of these measures is lacking. |
| 6.2 Effectiveness of current management practices in place based on surveillance data or other sources of information | | L | Evaluation of the effectiveness of risk management interventions is needed. WGS can be valuable in determining the impact of farming practices on AMR, virulence, and survival. It would also be useful for source attribution and control in the processing and distribution chain |
| N/A – not applicable; *Data quality score: data were only scored as it pertains to risk, background information were not scored; #: Y-Yes, N-No, S-Some, L-Limited | | | |

**Supplementary material Table S2**

**Frequency of *E. coli* recovery and CFU concentrations in water, sediment, and seafood product at farm, processing, depot, and retail**

|  |  |  | **Sample Prevalence (%) and/or concentrations (CFU)** | | | | |
| --- | --- | --- | --- | --- | --- | --- | --- |
| **Year of study/publication** | **Level in the food-chain** | **Country** | **Water** | **Sediment** | **Shrimp** | **Salmon**  **(fish)** | **Reference** |
| 1982-1983/1985 | Retail | England (source countries not specified) | - | - | Whole - 2 (n=148)  @1.0-1.99 log_10_ CFU/g  Peeled – 1 (n=148)  @2.0-2.99 log_10_ CFU/g | - | [1] |
| NR/1994 | Retail | Brazil (Domestic source) | - | - | 33.7-40% (n=30)  Range 10^0^->10^4^ CFU/g | - | [2] |
| NR /1994 | Retail | Bangladesh (Domestic source) | - | - | 1.2x10^3^ (eggs) - 1.2x10^5^ CFU/g (swimmerets) |  | [3] |
| 1994-1995/1998 | Processing unit | India | - | - | 2%  (n=1264 – raw shrimp) | - | [4] |
| 1998-2001/2005 | Ocean  Farm  Processing  Retail | Indonesia | -  <1-4.0 log_10_ CFU/g  -  - | -  -  -  - | <1 -3.8 log_10_ CFU/g  <1-4.0 log_10_ CFU/g  0-2.9 log_10_ CFU/g  <1-3.5 log_10_ CFU/g | -  -  - | [5] |
| 1998-1999/2003 | Processing unit | India (Domestic source) | - | - | 1.3-4.8% (n=2210) | - | [6] |
| NR/2004 | Processing unit | India | - | - | Whole prawns 50%  Headless prawns 25%  (n=18)  <20 CFU/g |  | [7] |
| NR/2005 | Processing  Landing centre  Retail | India | -  -  66 (n=12) | -  -  - | 8.6% (n=23)  16% (n=25)  15% (n=20) | -  - | [8] |
| 2001-2003/2005 | Farm | Southeast Asia (2 countries)  Central Asia (1)  Central America (1)  North America (1)  Pacific Ocean (1) | <100/100 ml or g  92.06%  100–1,000/100 ml or g  4.37%  >1,000/100 ml or g  3.57%  (n=252) | <100/100 ml or g  87.17%  100–1,000/100  ml or g  7.08  >1,000/100 ml or g  5.75%  (n=226) | <100/100 ml or g  80.57%  100–1,000/100 ml or g  12.55%  >1,000/100 ml or g  6.88%  (n=247) | - | [9] |
| 2005-2006/2008 | Freshly caught | Nigeria | -  - | -  - | 3.35x10^4^_CFU/ml_  _(M. vollenhovenii)_  5.56x10^4^_CFU/ml_  _(P.atlantica)_ | - | [10] |
| 2009-2010/2011 | Retail | United States (Imported products from China (n=83), Chile (n=34), Thailand (n=24), Canadian and Ecuador (n=8 each), Indonesia (n=5), Mexico (n=3) Bangladesh (n=2), and Honduras, India, Norway, and Vietnam (n=1 each) | - | - | 10.5 (n=38)  (Thailand (2), others (2)) | 4.8 (n=63)  (Chilean salmon) | [11] |
| 2007-2008/2012 | Retail | Brazil |  |  |  | <3.0-4.6x10^2^ MPN/g  (thermotolerant coliforms)  (n=31) | [12] |
| NR/2013 | Farm  Retail | India | - | - | 48.3% (n=60)  40% (n=40) | -  - | [13] |
| 2006-2007/2014 | Farm | Bangladesh | 8.4-14.4 (n=NR^a^) 408-1034 CFU/ml | 82.9-87.7 (n=NR)  4303-6056 CFU/g | 2.7-3.9% (n=NR)  189-196 CFU/g | - | [14] |
| NR/2015 | Retail, fishing harbor | India |  |  | MPN 9.5/g  (fecal coliforms) |  | [15] |
| NR/2016 | Farm  Depots | Bangladesh | 62.5 (n=16)  - | 43.7 (n=16)  - | 60.5 (n=30)  53.3% (n=45) | -  - | [16] |
| NR/2016 | Retail | India (source countries not specified) | - | - | 2%  (n=50) | - | [17] |
| NR/2016 | Retail | Germany | - | - | 31.3% (fresh)  12.5% frozen  2.59 (fresh)  log CFU/g  2.7 (frozen)  log CFU/g  (n=16) | 0-23.8% (fresh)  0-4.8 %(frozen)  <2.0-2.2(fresh)  log CFU/g  <2.0-2.3 (frozen) log CFU/g  (n=21) | [18] |
| NR/2016 | Retail | Czech Republic (source countries not specified) | - | - | - | 3.5x10^1^-4.5x10^4^ CFU/g  (1.6-1.7 log_10_ CFU/g) | [19] |
| 2006-2007/2016 | Retail | United States (National products) | - | - | - | 1.5% (combined)  (internet source, n=34)  (local source, n=32) | [20] |
| NR/2020 | Farm | Egypt | **Fishpond water inlets**  n=30  9/30 – 30%  **Tap water**  22/44 – 50%  n=44  **Outlet water**  n=26  12/26 – 46% | - | - | Fish  n=105  45/105 – 43% | [21] |

**Supplementary material Table S3**

**Antimicrobial resistance in *Escherichia coli* in wild and cultured aquatic animals and their environment**

| **Source** | **Country** | ***E. coli* prevalence**  **n/N (%)** | **Resistance phenotype (s) identified** | **Resistance genotype(s) identified** | **Associated genetic element** | **Ref.** |
| --- | --- | --- | --- | --- | --- | --- |
| Ready to eat shrimp | USA | 8/13 (62)  (Incidence) | AMP, CRO. NA, TET, TMP | - | - | [22] |
| Water and mud from culture ponds | Vietnam | Isolates grown on ATB selective media | NAL, SMX,TMP | - | - | [23] |
| Retail fish, shrimp, shellfish, processing plants, landing centers | India | Overall 73/188 (39)  Shrimp 9/68 (13) | AM, AMX, CEP, CRO, KAN, NA, PEN, STR, TET, VAN | - | Plasmids present | [24] |
| Farmed catfish | Vietnam | 11 *E. coli* isolates from pooled samples | AMP, CHL, OTC, SXT | - | - | [25] |
| Water, sediment, shrimp, fresh and saltwater fish | Malaysia, Thailand, Vietnam | Water, 39.7%  Sediment 28.8%  Fish 54.6% | CHL | - | - | [26] |
| Wild and imported shrimp | USA | 1/9 (wild)  0/13 (import) | AMP | - | - | [27] |
| Retail shellfish | Vietnam | 20 isolates selected from 50 shellfish samples | AMP, AMX/AMC, CEP, CIP, TET, CHL, ENR, GEN, KAN, NAL, NOR, SUL, TMP, NOR, STR | aadA, aphA-1, cat1, cmlA, dhfrV, sulI, , TEM, Tet(A,B) | - | [28] |
| Catfish (Ictalurus punctatus) | USA | 63 Isolates examined from enriched media | AMP, PEM, STR, BAC, RIF, CHL, SXT | dfrA12, dfrA17, aadA5 , aadA2, tetA, tetB, tetC | Integrons | [29] |
| Fish landing, processing and retail | India | 48/48 (100) | AMP, AMX, CIP, CEP, CHL, CRO NA, STR, TET, RIF | - | - | [30] |
| Shrimp, pond sediment and water | Brazil | - | AMP, CEP, GEN, **IMP**, NIT, SXT, TET | - | - | [31] |
| Retail, raw fish and RTE fish | India | 21 STEC isolates  2 non-STEC isolates from 54 raw and 27 RTE fish samples | AN, AMX, AMP, CEC, CTX, CHL, CIP, COL, SXT, ERY, GEN, KAN, LZD, NOR,OFX, PEN, PMB, STR TET, TMP | *tetA, tetB, strA, strB* | - | [32] |
| Imported seafood | Chile, Canada, China, Norway (Salmon)  Indonesia, Thailand(Shrimp) | Salmon 3/63 (4.8)  Shrimp 4/38 (10.5) | AMP, SXT, TET | - | - | [11] |
| Wholesale and retail seafood | Korea | 179/2662 (6.7%) | AMP, CAZ, CEP, CHL, CIP, GEN, KN, NA, SAM, STR, SXT/TMP, TET, TIC | Tet(A,B,D), aadA, TEM | Integron (class1 and 2)  dfrA12- aadA2, aadB | [33] |
| Retail fish (fish gut samples) | China | 218/300 (73) fish gut samples | AMP, CIP, CTF, CTX, CHL, FFC, SPC, KAN, NA, TET, SMX/TMP | Qnr, aac(6′ )-Ib-cr | Plasmids and gene co-transfer | [34] |
| River water | Portugal | Isolate grown on imipemen supplemented media | AMC, AMX, AN, ATM, CAZ, CTX, CXM, **ETP**, FEP, FOX, CIP, **IMP**, GEN, **MEM**, PIP, TIC | **blaKPC-2** | Plasmid, transposon Tn4401a | [35] |
| Retail salmon | Brazil |  |  |  |  | [12] |
| Fish farms Catfish (Clarias gariepinus) | Nigeria | 17 isolates from 90 samples | AMP, AMX, CHL, ERY, GM, NA, NOV, NIT, STR, TET | - | - | [36] |
| Retail shrimp, fishing harbor | India | MPN 9.5/g | AMP, GEN, PEN, TET, NIT |  |  | [15] |
| Retail shrimp | Vietnam | Isolates grown on cefoxatime supplemented media | AMP, CAZ, CHL, CIP, CTX, FOF, GEN, KAN, NAL, SXT/TMP, STR, TET | CTX-M, SHV, TEM | - | [37] |
| Retail shrimp | Switzerland | Salmon 3/11 (27)  Shrimp 7/11 (64) | AMP, CIP, CHL, NAL, SMX, TET, TMP | - | - | [38] |
| Retail seafood | India | Finfish 1/14 (7)  Shellfish 0/5 | CAZ, CIP, CRO, CTX, ETP, FEP, IMP, LVX, MEM, PIP/TZB | SHV, TEM, CTX-1, CTX-25, OXA-1, **NDM-5** | - | [39] |
| River water | Portugal | Isolate grown on imipemen supplemented media | All β-lactams, fluoroquinolones, and aminoglycosides (except amikacin) | bla_VIM-1,_ bla_VIM-34,_ bla_IMP-8_ | IncFIB plamid | [40] |
| Hospital food, cooked and raw fish | Iran | Raw, 1/70  Cooked 3/110 | AMP, AN, CAZ, CRO, FEP, GEN, MEZ, PMB, SMX, SXT, TET, TMP, VAN | tetA, dfrA1, aac (3)-IV, CITM | - | [41] |
| Retail seafood, venus clam | Germany | 1 *E. coli* isolate from 45 screened Enterobacteriaceae | CPM (other ARG’s derived from whole genome sequence) | *aac*A4-like, *aad*A1, *aph*(3‘)-XV, *bla*ACC-1, *bla*SHV-12, ***bla*VIM-1**, *cat*B2, *dfr*A14-  like, *mph*(A), *qnr*S1, *str*A-like, *str*B-like, *sul*1, *sul*2 | IncY plasmid | [42] |
| Retail and wholesale fish |  | 9/136 (*East1, EPEC, EAEC)* | AMC, AMP, CEP, FOX, NAL, TET | - | - | [43] |
| Retail fish, shrimp and seafood | India | 19 seafood samples, Enterobacteriaceae  ESBL+(169/215) | CAZ, CTX, CPD, IPM, ETP, FOX, MRP, CIP, ATM, AMC, TZP | blaCTX blaSHV blaTEM **blaNDM** | - | [44] |
| Wild and cultured fish stools | Brazil | STEC and EPEC screening of fish samples in BHI | AMP, CFL, TET |  |  | [45] |
| Effluent from shrimp farms | Vietnam | 1 Isolate grown on ATB selective media | - | sul1, sul2, qnrA, ermB | Plasmids? | [46] |
| Retail seafood, shrimp and shellfish | Germany (several sources some unknown?) | - | ESBL+ | blaSHV, þblaACC, blaCTX-M, blaTEM, blaCMY-2 | - | [47] |
| Retail fish | Cambodia | ESBL+ *E. coli*; 32 (53%) of 60  Fish | AMG, APL, COL, CPM, ESBL+, FLQ, MAC, SXT, TET | AMG (aad, aac, aph, strA/B), APL (cat, floR, cmlA), COL (mcr), CPM (*bla*OXA-181), ESBL (blaCTX-M, blaCMY-2)FQL (qnrSq, *aac(6')Ib-cr*, *oqxA*), MAC(erm,mph mef, Inu), SXT (SUl, dfr), TET(A,B,M) | - | [48] |
| Farmed fish  Inlet;oulet water  Tap water | Egypt | 45/105 (43%)  9/30 (30%); 12/26 (46%)  22/44 (50%) | CAZ, CRO, CTX, ETP, FOX, IPM, MEM | *bla*_KPC_, *bla*_OXA-48_, and *bla*_NDM_  *bla*_CTX-M-15_, *bla*_SHV_, *bla*_OXA-1_, *bla*_TEM_, and *bla*_PER-1_ | plasmids | [21] |

**AMC** Amoxicillin-clavulanic acid, **AMG** Aminoglycosides, **AN** Amikacin, **AMP** Ampicillin, **AMX** Amoxicillin, **APL** Amphenicol, **AZM** Azithromycin, **ATM** Aztreonam, **BAC** Bacitracin, **CAZ** Ceftazidime, **CPM** carbapenem, **CXM** Cefuroxime, **CTX** Cefotaxime, **CEC** cefaclor, **CEP** Cephalothin, **FEP** Cefepime, **CPD** Cefpodoxime, **CHL** Chloramphenicol, **CIP** Ciprofloxacin, **CLA** Clavulanic acid; **CLI** Clindamycin, **COL** Colistin, **CRO** Ceftriaxone, **ETP** Ertapenem, **ERY** Erythromycin, **FIS** Sulfisoxazole, **FLQ** Fluoroquinolones, **FFC** Florfenicol, **FOF** Fosmycins, **FOX** Cefoxitin, **GEN** Gentamicin, **IMP** Imipenem, **KAN** Kanamycin, **LVX** levofloxacin, **LZD** linezolid, **MAC** Macrolides, **MEM** Meropenem, **MEM** Meropenem, **MEZ** Mezlocillin, **NAL** Nalidixic acid, **NIT** Nitrofurantoin, **NOR** norfloxacin, **NOV** Novobiocin, **OFX** ofloxacin, **PEN** Penicillins, **PIP** Piperacillin, **PMB** polymyxin B, **OTC** Oxytetracycline, **RIF** Rifampicin, **SMX** Sulfamethoxazole, **SPC** Spectinomycin, **SAM** Ampicillin/sulbactam, **SSS** Sulfisoxazole, **STR** Streptomycin, **SXT** Trimethoprim-sulfamethoxazole, **TZB** Tazobactam, **TBC** total bacterial counts, **TEL** Telithromycin, **TET** Tetracycline, **TIC** Ticarcillin.**TIO** Ceftiofur, **TMP** Trimethoprim, **VAN** Vancomycin

**References**

1. **Greenwood MH, *et al.*** The Microbiology of Cooked Prawns and Shrimps on Retail Sale Matthews and S . Patrick Published by : Cambridge University Press Stable URL : http://www.jstor.org/stable/3862959. 1985; **94**: 319–326.

2. **Ayulo, A M R, Machado, R A, Scussel VM**. Enterotoxigenic Escherichia coli and Staphylococcus aureus in fish and seafood from the southern region of Brazil Andres Mane R o m e r o Ayulo a , b ,* Ruben Abreu Machado b ,. *International journal of food microbiology* 1994; **24**: 171–178.

3. **Rahim Z, *et al.*** Enterotoxigenicity, Hemolytic Activity and Antibiotic Resistance of Aeromonas Spp. Isolated from Freshwater Prawn Marketed in Dhaka, Bangladesh. 1994; **38**: 773–778.

4. **Mohamed Hatha A., Paul N, Rao B**. Bacteriological quality of individually quick-frozen (IQF) raw and cooked ready-to-eat shrimp produced from farm raised black tiger shrimp (Penaeus monodon). *Food Microbiology* 1998; **15**: 177–183.

5. **Dewanti-Hariyadi R, Suliantari LN, Fardiaz S**. Distribution, frequency and concentrations of the AMR hazard(s) in the food chain. 2005; : 63–70.

6. **Mohamed Hatha A., Maqbool T., Suresh Kumar S**. Microbial quality of shrimp products of export trade produced from aquacultured shrimp. *International Journal of Food Microbiology* 2003; **82**: 213–221.

7. **Bandekar JR, *et al.*** Bacteriological quality of farmed freshwater fish and shellfish meant for export. *Fishery Technology* 2004; **41**: 57–62.

8. **Kumar HS, *et al.*** Prevalence and antibiotic resistance of Escherichia coli in tropical seafood. *World Journal of Microbiology and Biotechnology* 2005; **21**: 619–623.

9. **Koonse B, *et al.*** Salmonella and the Sanitary Quality of Aquacultured Shrimp. *Journal of Food Protection* 2005; **68**: 2527–2532.

10. **Bello-Olusoji O**. Bacteriological Studies of Some Prawns, Parapeneopsis atlantica and Macrobrachium vollenhovenii, Under Different Processing Methods. Bello-Olusoji O (correspondence author), ed. *Advances in Food Sciences* 2008; Published online: 2008.

11. **Wang F, *et al.*** Prevalence and antimicrobial susceptibility of major foodborne pathogens in imported seafood. *Journal of food protection* United States, 2011; **74**: 1451–1461.

12. **Nespolo NM, Martineli TM, Rossi Jr. OD**. Microbiological quality of salmon (Salmo salar) sold in cities of the state of São Paulo, Brazil. *Brazilian Journal of Microbiology* 2012; **43**: 1393–1400.

13. **Prakash M, Karmagam N**. A study on bacterial flora associated with fresh water prawn , Macrobrachium rosenbergii. *Int. J. Curr. Res. Aca Rev.* 2013; **1**: 1–16.

14. **Lekshmy S, *et al.*** Incidence of E. coli in extensive shrimp culture systems of Kerala. 2014; **9**: 117–126.

15. **Chakravarty MS, *et al.*** *Escherichia coli-occurrence in the meat of shrimp, fish, chicken and mutton and its antibiotic resistance*. *Pelagia Research Library European Journal of Experimental Biology*. 2015.

16. **Faridullah M, Roy VC, Lithi UJ**. Prevalence of Salmonella and Escherichia coli contamination in shrimp (Penaeus monodon) farms, depots and processing plants in different areas of Bangladesh. *Asian Journal of Medical and Biological Research* 2016; **2**: 171–176.

17. **Surendraraj A, Thampuran N, Joseph TC**. Molecular Screening, Isolation, and Characterization of Enterohemorrhagic Escherichia coli O157:H7 from Retail Shrimp. *Journal of Food Protection* 2010; **73**: 97–103.

18. **Atanassova V, Reich F, Klein G**. Microbiological Quality of Sushi from Sushi Bars and Retailers. *Journal of Food Protection* 2008; **71**: 860–864.

19. **Cwiková O**. Microbiological evaluation of fish. *Potravinarstvo* 2016; **10**: 407–412.

20. **Pao S, *et al.*** Microbial Quality of Raw Aquacultured Fish Fillets Procured from Internet and Local Retail Markets. *Journal of Food Protection* 2008; **71**: 1544–1549.

21. **Hamza D, *et al.*** Emergence of β-lactamase- and carbapenemase- producing Enterobacteriaceae at integrated fish farms. *Antimicrobial Resistance and Infection Control* 2020; **9**: 1–12.

22. **Duran GM, Marshall DL**. Ready-to-Eat Shrimp as an International Vehicle of Antibiotic-Resistant Bacteria. *Journal of Food Protection* 2005; **68**: 2395–2401.

23. **Le TX, Munekage Y, Kato SI**. Antibiotic resistance in bacteria from shrimp farming in mangrove areas. *Science of the Total Environment* 2005; **349**: 95–105.

24. **Kumar HS, *et al.*** Prevalence and antibiotic resistance of Escherichia coli in tropical seafood. *World Journal of Microbiology and Biotechnology* 2005; **21**: 619–623.

25. **Sarter S, *et al.*** Antibiotic resistance in Gram-negative bacteria isolated from farmed catfish. *Food Control* 2007; **18**: 1391–1396.

26. **G. H, *et al.*** *Biodiversity of chloramphenicol-resistant mesophilic heterotrophs from Southeast Asian aquaculture environments*. *Research in Microbiology*. 2007.

27. **Boinapally K, Jiang X**. Comparing antibiotic resistance in commensal and pathogenic bacteria isolated from wild-caught South Carolina shrimps vs. farm-raised imported shrimps. *Canadian Journal of Microbiology* 2008; **53**: 919–924.

28. **Van TTH, *et al.*** Safety of raw meat and shellfish in Vietnam: An analysis of Escherichia coli isolations for antibiotic resistance and virulence genes. *International Journal of Food Microbiology* 2008; Published online: 2008.doi:10.1016/j.ijfoodmicro.2008.03.029.

29. **Nawaz M, *et al.*** Molecular Characterization of Tetracycline-Resistant Genes and Integrons from Avirulent Strains of Escherichia coli Isolated from Catfish . *Foodborne Pathogens and Disease* 2009; **6**: 553–559.

30. **Kumaran S, *et al.*** Antibiotic resistant Esherichia coli strains from seafood and its susceptibility to seaweed extracts. *Asian Pacific Journal of Tropical Medicine* Hainan Medical College, 2010; **3**: 977–981.

31. **Vieira RHSDF, *et al.*** Antimicrobial susceptibility of Escherichia coli isolated from shrimp ( Litopenaeus vannamei ) and pond environment in northeastern Brazil. *Journal of Environmental Science and Health, Part B* 2010; **45**: 198–203.

32. **Rao TS, *et al.*** Multi drug resistance patterns of shiga toxin - producing Escherichia coli (STEC) and non - STEC isolates from meats, RTE meat foods, drinking water and human diarrhoeic samples of Punjab, India. *Archives of Clinical Microbiology* 2011; **2**: 1–12.

33. **Ryu SH, *et al.*** Antimicrobial resistance and resistance genes in Escherichia coli strains isolated from commercial fish and seafood. *International Journal of Food Microbiology* 2012; Published online: 2012.doi:10.1016/j.ijfoodmicro.2011.10.003.

34. **Jiang HX, *et al.*** Prevalence and characteristics of β-lactamase and plasmid-mediated quinolone resistance genes in Escherichia coli isolated from farmed fish in China. *Journal of Antimicrobial Chemotherapy* 2012; **67**: 2350–2353.

35. **Poirel L, *et al.*** Environmental KPC-Producing Escherichia coli Isolates in Portugal. *Antimicrobial Agents and Chemotherapy* 2012; **56**: 1662–1663.

36. **Efuntoye MO, Olurin KB, Jegede GC**. Bacterial flora from healthy clarias gariepinus and their antimicrobial resistance pattern. *Advance Journal of Food Science and Technology* 2012; Published online: 2012.

37. **Le QP, *et al.*** Characteristics of Extended-Spectrum B-Lactamase Producing Escherichia coli in Retail Meats and Shrimp at a Local Market in Vietnam. *Foodborne Pathogens and Disease* 2015; **12**: 719–725.

38. **Boss R, Overesch G, Baumgartner A**. Antimicrobial Resistance of Escherichia coli, Enterococci, Pseudomonas aeruginosa, and Staphylococcus aureus from Raw Fish and Seafood Imported into Switzerland. *Journal of Food Protection* 2016; **79**: 1240–1246.

39. **Singh AS, *et al.*** Isolation of Escherichia coli harboring blaNDM-5 from fresh fish in India. *Journal of Microbiology, Immunology and Infection* 2016; **49**: 822–823.

40. **Kieffer N, *et al.*** *VIM-1, VIM-34, and IMP-8 Carbapenemase-Producing Escherichia coli strains recovered from a Portuguese river*. *Antimicrobial Agents and Chemotherapy*. 2016.

41. **Ranjbar R, *et al.*** Shiga (Vero)-toxin producing Escherichia coli isolated from the hospital foods; virulence factors, o-serogroups and antimicrobial resistance properties. *Antimicrobial Resistance & Infection Control* Antimicrobial Resistance & Infection Control, 2017; **6**: 4.

42. **Roschanski N, *et al.*** VIM-1 carbapenemase-producing Escherichia coli isolated from retail seafood, Germany 2016. *Eurosurveillance* European Centre for Disease Prevention and Control (ECDC), 2017; **22**: 17–32.

43. **Wang L, *et al.*** Prevalence, antimicrobial resistance and multiple-locus variable-number tandem-repeat analysis profiles of diarrheagenic Escherichia coli isolated from different retail foods. *International Journal of Food Microbiology* Elsevier B.V., 2017; **249**: 44–52.

44. **Lekshmi M, *et al.*** Multiple Antibiotic-Resistant, Extended Spectrum-β-Lactamase (ESBL)-Producing Enterobacteria in Fresh Seafood. *Microorganisms* 2017; **5**: 53.

45. **Cardozo MV, *et al.*** Shigatoxigenic and atypical enteropathogenic Escherichia coli in fish for human consumption. *Brazilian Journal of Microbiology* 2018; **49**: 936–941.

46. **Pham TTH, *et al.*** Analysis of antibiotic multi-resistant bacteria and resistance genes in the effluent of an intensive shrimp farm (Long An, Vietnam). *Journal of Environmental Management* 2018; **214**: 149–156.

47. **Vu TTT, *et al.*** Investigation of Extended-Spectrum and AmpC β-Lactamase–Producing Enterobacteriaceae from Retail Seafood in Berlin, Germany. *Journal of Food Protection* 2018; Published online: 2018.doi:10.4315/0362-028x.jfp-18-029.

48. **Nadimpalli, Maya, Vuthy, Yith , Lauzanne A De, *et al.*** Meat and Fish as Sources of Extended-Spectrum β-Lactamase– Producing Escherichia coli, Cambodia. *Emerging Infectious Diseases* 2019; **25**: 1–6.
